# Genetic basis of high aroma and stress tolerance in the oolong tea cultivar genome

**DOI:** 10.1038/s41438-021-00542-x

**Published:** 2021-05-01

**Authors:** Pengjie Wang, Jiaxin Yu, Shan Jin, Shuai Chen, Chuan Yue, Wenling Wang, Shuilian Gao, Hongli Cao, Yucheng Zheng, Mengya Gu, Xuejin Chen, Yun Sun, Yuqiong Guo, Jiangfan Yang, Xingtan Zhang, Naixing Ye

**Affiliations:** 1 College of Horticulture, Fujian Agriculture and Forestry University/Key Laboratory of Tea Science in Universities of Fujian Province, 350002 Fuzhou, China; 2 Shenzhen Branch, Guangdong Laboratory for Lingnan Modern Agriculture, Genome Analysis Laboratory of the Ministry of Agriculture, Agricultural Genomics Institute at Shenzhen, Chinese Academy of Agricultural Sciences, 518120 Shenzhen, China; 3 Center for Genomics and Biotechnology, Fujian Provincial Key Laboratory of Haixia Applied Plant Systems Biology, Key Laboratory of Genetics, Fujian Agriculture and Forestry University, 350002 Fuzhou, China

**Keywords:** Genomics, Plant genetics

## Abstract

Tea plants (*Camellia sinensis*) are commercially cultivated in >60 countries, and their fresh leaves are processed into tea, which is the most widely consumed beverage in the world. Although several chromosome-level tea plant genomes have been published, they collapsed the two haplotypes and ignored a large number of allelic variations that may underlie important biological functions in this species. Here, we present a phased chromosome-scale assembly for an elite oolong tea cultivar, “Huangdan”, that is well known for its high levels of aroma. Based on the two sets of haplotype genome data, we identified numerous genetic variations and a substantial proportion of allelic imbalance related to important traits, including aroma- and stress-related alleles. Comparative genomics revealed extensive structural variations as well as expansion of some gene families, such as terpene synthases (TPSs), that likely contribute to the high-aroma characteristics of the backbone parent, underlying the molecular basis for the biosynthesis of aroma-related chemicals in oolong tea. Our results uncovered the genetic basis of special features of this oolong tea cultivar, providing fundamental genomic resources to study evolution and domestication for the economically important tea crop.

## Introduction

The development of high-throughput sequencing has vigorously promoted research progress in plant genomics and genetics, especially single-molecule long-read sequencing and high-throughput chromatin conformation capture (Hi-C) technology, which have allowed complete plant genome sequencing and assembly at a low cost^1^. Diploid or polyploid genomes consist of two or more homologous chromosome sets. Notably, most plant reference genome assemblies are collapsed homologous copies of each chromosome. Although such assembled genomes are convenient for comparison and inference, they ignore allelic variants that may have potential biological functions and affect the quality of heterozygous plant genomes^2^. Unzipping haplotypes into two or more sequence sets has become a new trend in genome assembly that is conducive to research on plant heterosis and evolution and provides a solid foundation for accurate and reliable genome editing. ALLHiC is a new algorithm for constructing allele-aware, chromosome-scale assemblies based on Hi-C data^3^, and it has been successfully applied to the genome assembly of several plant species, including an autotetraploid^4^ and an autooctoploid^3^ sugarcane genome, an autotetraploid cultivated alfalfa genome^5^, an allotetraploid rapeseed genome^6^, and two Ficus genomes^7^. ALLHiC is sensitive to the quality of input assembly and becomes powerless when the proportion of chimeric contigs and collapsed regions increases. However, high-precision sequencing technology and advanced assembly algorithms will help ALLHiC cross this barrier. Therefore, ALLHiC is a powerful tool for genome research on heterozygous diploid and polyploid plants.

The tea plant *Camellia sinensis* L. O. Kuntze is a perennial evergreen woody plant that is commercially cultivated in >60 countries and accounts for >5 million tons of leaves picked each year^8^. At present, >2 billion cups of tea are consumed worldwide every day. These teas are mainly manufactured by processing fresh tea leaves and are rich in characteristic secondary compounds, including theanine, caffeine, catechins, and volatiles, which are beneficial to human health^9^. The United Nations General Assembly (UNGA) designated May 21 as “International Tea Day” to praise the high value of tea to the global society, economy, and culture, especially its significant role in rural poverty reduction and development in developing countries. However, compared with the tremendous contribution of tea to the global economic industry, fundamental biological research on tea plants and breeding efficiency are still lagging^10^.

Tea plants are an ideal model for investigating the molecular regulation mechanism of secondary metabolites^11^. The rich collection of secondary metabolites endow tea with a distinctive taste, aroma and color and are involved in the responses to various environmental stresses^12,13^; thus, these metabolites are attracting increasing research attention. Great advances in tea genomics have effectively facilitated researchers’ understanding of tea quality and adaptability. The genomic sequences of two major tea plant varieties, namely, *Camellia sinensis* vars. *sinensis* (CSS)^9,14–17^ and *assamica* (CSA)^18^ have been resolved, especially those of three CSS cultivars (cultivars Shuchazao^9,15^, Biyun^16^, and Longjin 43^17^) whose high-precision chromosome-level genomic sequences have been obtained. In addition, the high-quality genome of an ancient tea tree has been published^19^. However, these genomic assemblies have collapsed haplotypes and ignore the large number of allelic variations that exist in the highly heterozygous tea genome.

In general, tea plants exhibit self-incompatibility^20^, which leads to high levels of heterozygosity and allelic variation in their genomes. The allelic variations between two haplotypes within a single genome may play a crucial role in phenotypic variation, heterosis, and evolution^2,3^. Several studies support the key point that hybrid rice exhibits heterosis owing to alleles with high transcriptional activity and dominant expression^21,22^. In this study, we generated a chromosome-scale reference genome of CSS Huangdan (HD, 2*n* = 2*x* = 30) by PacBio HiFi (high-fidelity) sequencing and Hi-C technology. A phased genome with two fully represented haplotypes was also generated to facilitate the mining of allelic variation and allele-specific expression (ASE). HD is an elite tea cultivar originating from Anxi County, Fujian Province, China. Commercially, HD is suitable for processing into oolong, green, and black teas and was approved by the Chinese Crop Variety Approval Committee in 1985 (No. GS13008-1985). HD is not only a standard reference species for breeding oolong tea cultivars but also a high-aroma backbone parent that has been used to breed a series of excellent hybrid offspring^23,24^, including CSS Jinguanyin (JGY, No. GS2002017), CSS Huangguanyin (HGY, No. GS2002015), and CSS Jinmudan (JMD, No. GS2010024). Our research will enhance the understanding of genetic variation and heterosis of tea plants and lay the foundation for precise gene editing and molecular breeding in the future.

## Results

### Genome assembly and annotation

**Fig. 1:**
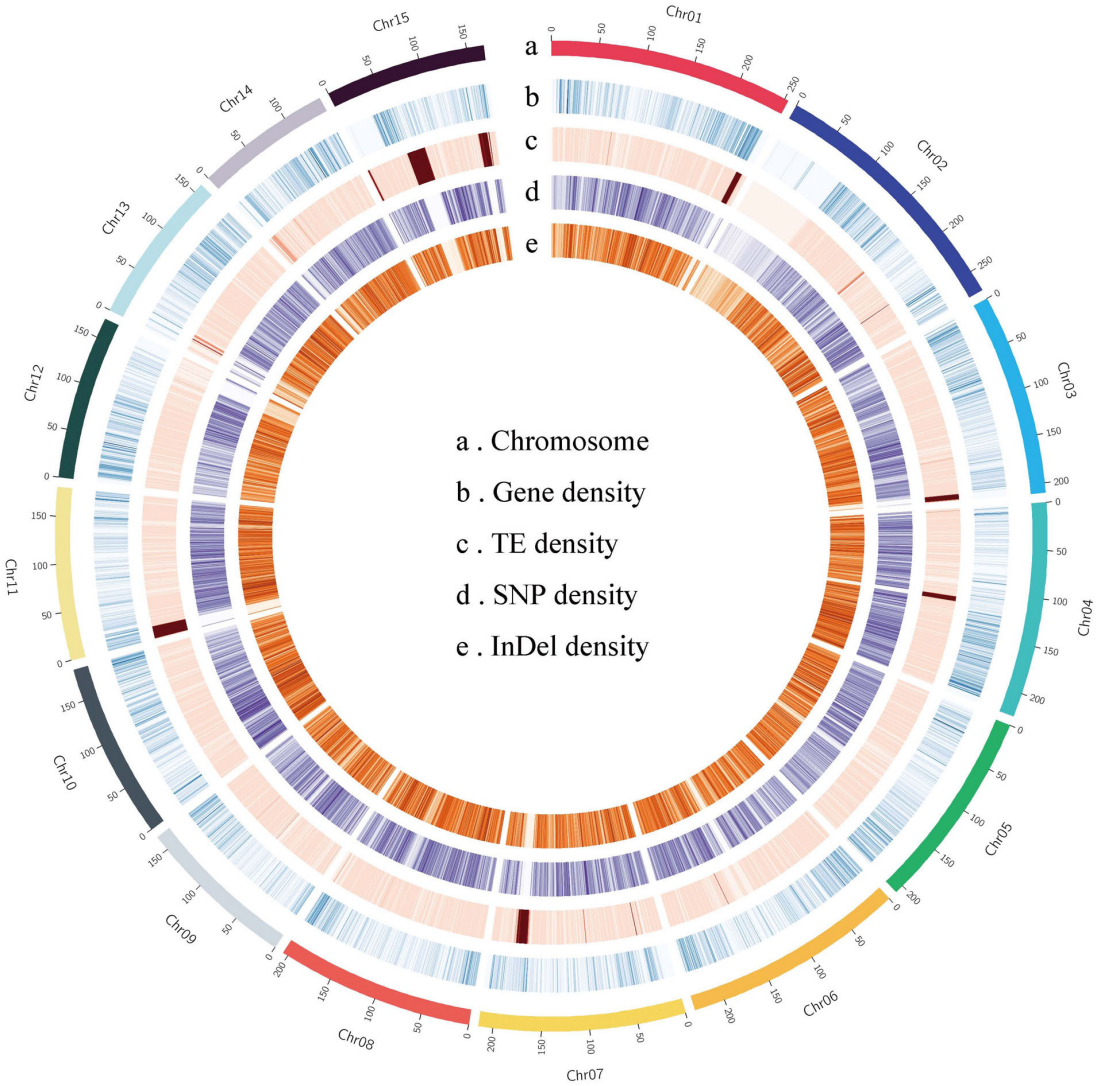
Genomic features of the HD genome along 15 pseudochromosomes. The tracks from outermost to innermost indicate **a** pseudochromosomes of the monoploid genome, **b** gene density, **c** transposable element (TE) density, **d** single-nucleotide polymorphism (SNP) density, and **e** insertion-deletion (InDel) density

**Fig. 2:**
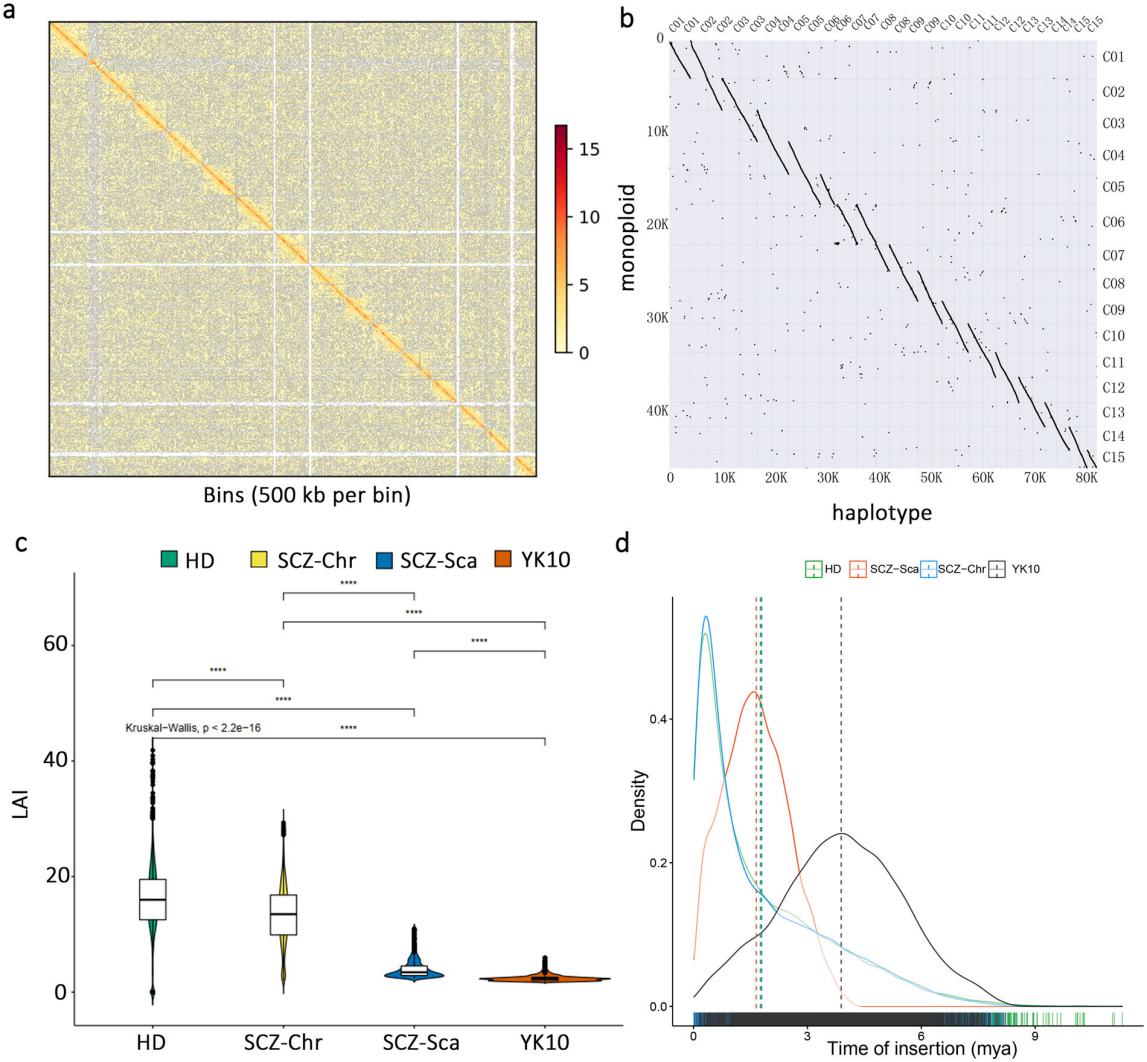
Assembly and LTR features of HD genome. **a** Chromatin interaction at 500-Kb resolution in the HD genome. **b** Syntenic analysis of HD monoploid genome assembly and HD-phased haplotypes. **c** Assessment of assembly quality with LAI value, where the *y*-axis represents LAI. **d** Distribution of LTR insertion time in HD and three published tea plant genomes. Dashed lines represent the mean insertion time in each genome

A total of 54.90 Gb (~18.67-fold) of high-fidelity (HiFi) PacBio reads were sequenced on the PacBio Sequel II platform. In addition, chromosomal-level genome assembly was achieved using 327.80 Gb (~111.50-fold) of Illumina short reads generated from two high-throughput conformation capture (Hi-C) libraries (Supplementary Table 1). To overcome the assembly problem caused by the high level of heterozygosity (2.79–3.40%) of the tea plant genome, we generated nonredundant genome sequences using Hifiasm^25^ and Purge_Dups^26^, yielding a contig-level assembly with a total size of 2.94 Gb. The assembled genome size was consistent with the genome size estimated using flow cytometry (Supplementary Table 2). Compared with early published tea plant genomes, the contig N50 of the HD genome was 2.61 Mb, which is ~4.3-fold, ~38.1-fold, and ~127.7-fold that of chromosome-level CSS-Shuchazao (SCZ-Chr)^15^, scaffold-level CSS-Shuchazao (SCZ-Sca)^14^, and CSA-Yunkang 10 (YK 10)^18^, respectively (Table 1). BUSCO^27^ analysis showed that the HD contig set contained 95.0% complete core orthologous genes in plants (Supplementary Table 3). Subsequently, we anchored the nonredundant contig set onto 15 pseudochromosomes based on Hi-C contacts, representing a high-quality chromosome-scale monoploid genome assembly (Fig. 1). The length of the pseudochromosomes ranges from 140 Mb (Chr11) to 247 Mb (Chr1) (Supplementary Table 4). The interaction signals were enriched in chromosomes, and the intensity of interaction along the diagonal was relatively smooth, showing well-organized contig orderings (Fig. 2a). Assembly assessment through long-terminal repeat (LTR) annotation showed that the LTR Assembly Index (LAI) score^28^ of the HD monoploid assembly was 16.6 (Table 1 and Fig. 2c), indicating that more LTRs were recovered in our assembly than in other published tea genomes (Supplementary Table 5).

**Table 1:** Comparison of contig assemblies among tea plant genomes

	HD	SCZ-Chr	SCZ-Sca	YK 10
Contig assembly size (Gb)	2.94	2.94	2.89	2.57
Max length (Mb)	20.56	2.89	0.54	0.26
Contig N50 (kb)	2610.56	600.46	67.07	19.96
Complete BUSCO ratio (%)	95.0%	90.6%	93.1%	90.2%
Raw LAI	13.75	12.35	1.93	2.32
LAI	16.16	14.19	3.89	5.17
GC content (%)	38.82	38.25	37.84	42.31
No. of genes	43,779	50,525	33,932	36,951
Average gene length (bp)	5452	4906	6821	3290
Average CDS length (bp)	1092	1086	1345	990
Average exon length (bp)	342	245	238	237
Average exon number per gene	4.4	5.1	5.7	4.8
Exon GC content (%)	43.26	43.83	44.50	44.55
Average intron length (bp)	1223	973	1298	640
Intron GC content (%)	34.57	35.38	33.94	33.50

Although high heterozygosity presents difficulties in resolving the tea plant genome, it stores abundant genetic resources that the monoploid genome cannot decipher. The recent emergence of HiFi reads and the Hifiasm algorithm help us to overcome the difficulties of phasing highly heterozygous genomes. On this basis, the ALLHiC algorithm^3^ aids in constructing a haplotype-resolved chromosome-level assembly by separating homologous chromosomes according to the strength of interaction signals. We finally divided the HD genome into two haplotypes (A and B) with the assistance of the monoploid reference assembly. The two haplotypes comprised a total of 30 pseudochromosomes with a total length of 5.88 GB (2.92 Gb for HA and 2.95 Gb for HB). To assess the quality of the two haplotypes, we first compared them with the aforementioned HD monoploid reference assembly, showing good collinearity (Fig. 2b). BUSCO analysis showed that 89.9% and 92.5% conserved single-copy genes were recalled from haplotype A and haplotype B, respectively, and a total of 95.1% were complete BUSCO genes (Table 2).

**Table 2:** Phased chromosome-scale assembly and annotation

	Haplotype A	Haplotype B
Length of chromosomes (Gb)	2.92	2.95
BUSCO completeness of assembly (%)	89.9	92.5
Number of genes	44,105	44,409
Number of genes with >2 alleles		7211
Number of genes with 2 alleles		19,723
Number of genes with 1 allele		1066
Total number of genes with alleles		26,000

Combining information such as orthologous proteins, de novo prediction and expression evidence, we annotated 43,779 protein-coding genes through the MAKER pipeline^29^, of which 98.20% (42,981/43,779) of the genes were supported by transcript evidence, and 49.85% (21,826/43,779) and 41.44% (22,139/43,779) genes could be functionally annotated by the GO and KEGG databases, respectively. The average length of the protein-encoding genes was 5452 bp. In addition, the average length of introns was 1223 bp with 35.85% GC content, and the average length of exons was 342 bp with 43.26% GC content (Table 1). We further used TEclass^30^, a transposable element (TE) automatic classification tool, to enhance the repeatMasker annotation result. Finally, 70.75% of the HD genome was annotated as repetitive sequences (2.08/2.94 Gb) (Supplementary Table 6), and LTR retrotransposons (LTR-RTs) belonged to the largest class of TEs, accounting for 48.64% of the genomes (1.43/2.94 Gb). Meanwhile, 24,741 intact LTR-RTs were identified in the HD genome using LTR_retriever^31^ (Supplementary Table 5). Similar to the SCZ-Chr genome^15^, the HD genome showed more abundant LTR-RTs than two previously published draft genomes assembled based on Illumina short reads^14,18^, indicating that long-read sequencing technology provided a better solution for TE annotation in the highly repetitive genome. We also observed a significant enrichment of LTR-RT insertion events in the HD and SCZ-Chr genomes (Fig. 2d), dating a recent LTR burst back to 0.6 mya in the tea plant.

### Alleles associated with stress tolerance and aroma-related components

**Fig. 3:**
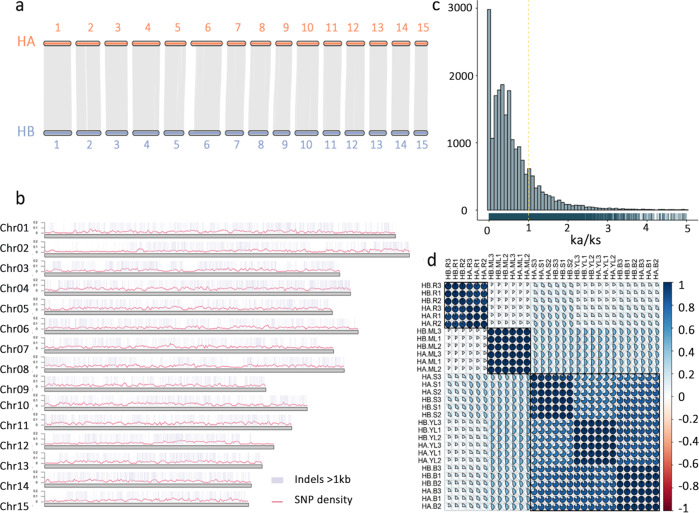
Allelic variations and expression between two HD haplotypes. **a** Synteny block between allele gene pairs. **b** Genetic variations between the two haplotypes. **c** Distribution of ka/ks between allele gene pairs. **d** Correlation analysis and clustering of the expression of alleles in five tissues, including roots (R), stems (S), buds (B), young leaves (YL), and mature leaves (ML)

Comparison between the two haplotypes revealed a high level of synteny (Fig. 3a). However, a large number of haplotypic variations were detected, including 23.57 million SNPs, 1.14 million insertions, and 1.13 million deletions (Supplementary Table 7). The genetic variants were widespread and evenly distributed across the 15 pseudochromosomes (Fig. 3b). The phased chromosome-scale genome assembly allows us to separate alleles from 59.38% (26,000/43,779) protein-coding genes with at least one base substitution. These alleles showed an average similarity of 92.6% and Ka/Ks ratio of 0.61 (Fig. 3c), suggesting relatively rapid evolution. Functional annotation revealed that the genes under positive selection (Ka/Ks>1) were enriched in multiple biological processes, such as the response to reactive oxygen species (ROS) and the metabolism of multiple terpenoids (Supplementary Fig. 1), indicating their important roles in responses to stress and likely association with aroma-related volatile organic compounds (VOCs). RNA-seq reads from five different tissues, including buds, roots, stems, young leaves, and mature leaves, were also mapped against the allelic genes from HA and HB. We evaluated and clustered the correlation between the expression of 26,000 allelic genes in five tissues and found that the expression of these alleles in the same tissue was highly correlated and clustered together (Fig. 3d).

### Extensive structural variations affected aroma-related genes

**Fig. 4:**
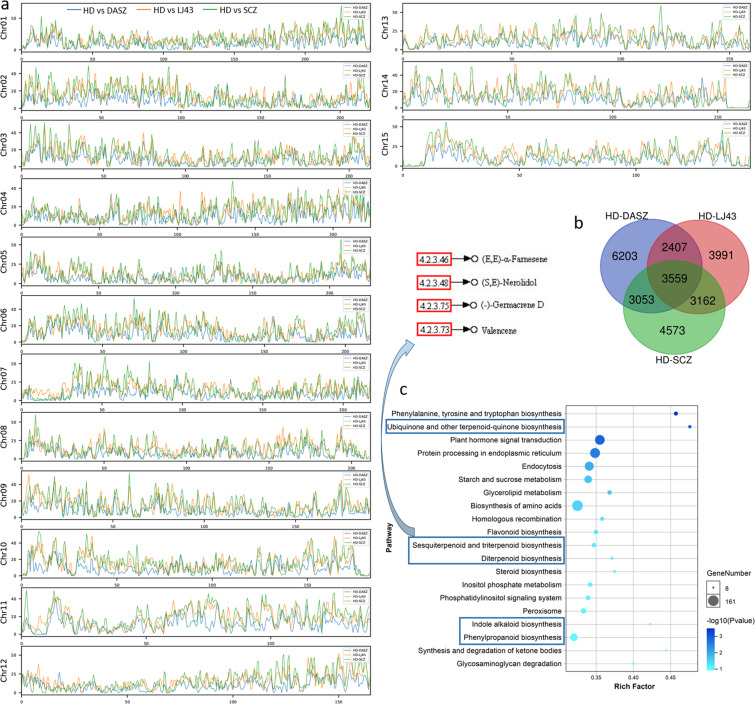
Analysis of SVs and related genes. **a** SV frequency plot of HD and three other tea plant genomes on 15 chromosomes. HD: Huangdan, SCZ: Suchazao^15^, LJ43: Longjing 43^17^, DASZ: An ancient tea tree^19^. **b** Venn plot of three sets of SV-annotated genes. **c** Top 20 KEGG pathways of SV-annotated genes between the HD and SCZ genomes. Pathways related to aroma are marked with blue boxes, and SV-related terpene synthases (TPSs) and their corresponding terpenoids are highlighted

Structural variants (SVs) represent genomic rearrangements greater than 50 bp, which are an important basis for crop improvement and domestication traits^32^. However, SVs have not yet been investigated in tea plants by comparing high-quality genomes. Here, we identified 45,304, 44,358, and 26,754 SVs by comparing HD to the three published SCZ-Chr^15^, LJ43^17^ and DASZ^19^ genomes (Table 3 and Fig. 4a). A similar level of SV was observed in the three genomes, ranging from 294.08 (9.77%) to 380.32 (12.63%) Mb. The SVs were not evenly distributed across the 15 chromosomes. For instance, chromosome 1 has a high SV frequency and the longest total length, and chromosome 12 has the lowest SV frequency (Fig. 4a). These SVs affected 14,347, 13,119, and 15,222 protein-coding genes in SCZ-Chr, LJ43 and DASZ, respectively, suggesting that the SVs had a large effect on gene function. Among these genes, 3559 were shared (Fig. 4b). These genes were involved in a variety of metabolic pathways in our KEGG analysis (Supplementary Fig. 2), including zeatin biosynthesis, starch and sucrose metabolism, amino acid biosynthesis, carotenoid biosynthesis, glycosphingolipid biosynthesis, and diterpenoid biosynthesis, indicating the metabolic differentiation of tea germplasm resources. While HD is suitable for high-aroma oolong tea, SCZ and LJ43 are famous for processing into green tea. We analyzed the SV annotation genes of HD vs. SCZ-chr and HD vs. LJ43 and found that these genes were annotated in many aroma-related pathways (Fig. 4c and Supplementary Fig. 3), which indicated that SVs may affect the high-aroma characteristics of HD. We also observed that many terpenoids, which are important components of the aroma of oolong tea, exhibited structural variations in their synthetase genes (Fig. 4c), including (*E*)-nerolidol synthase (NES) and α-farnesene synthase (AFS), which can be used as SV resources for high-aroma tea breeding.

**Table 3:** SV statistics of the HD genome vs. three tea plant genomes

	HD vs. SCZ	HD vs. LJ43	HD vs. DASZ
No. of InDels (50–500 bp)	8174	8351	3718
No. of InDels (500–10,000 bp)	4539	5132	2023
No. of InDels (10,000–50,000 bp)	456	400	246
No. of InDels (50,000–100,000 bp)	21	19	9
No. of tandem expansion/contraction	5209	4998	3653
No. of repeat expansion/contraction	26,905	25,458	17,105
Size of InDels (Mb)	24.21	24.17	11.64
Size of tandem expansion/contraction (Mb)	75.76	41.46	46.79
Size of repeat expansion/contraction (Mb)	280.36	254.62	235.65
Total SV number	45,304	44,358	26,754
Total SV size (Mb)	380.32	320.25	294.08
Gene count	14,347	13,119	15,222

### Expansion of the terpene synthase (TPS) family contributes to the high-aroma characteristics of HD

**Fig. 5:**
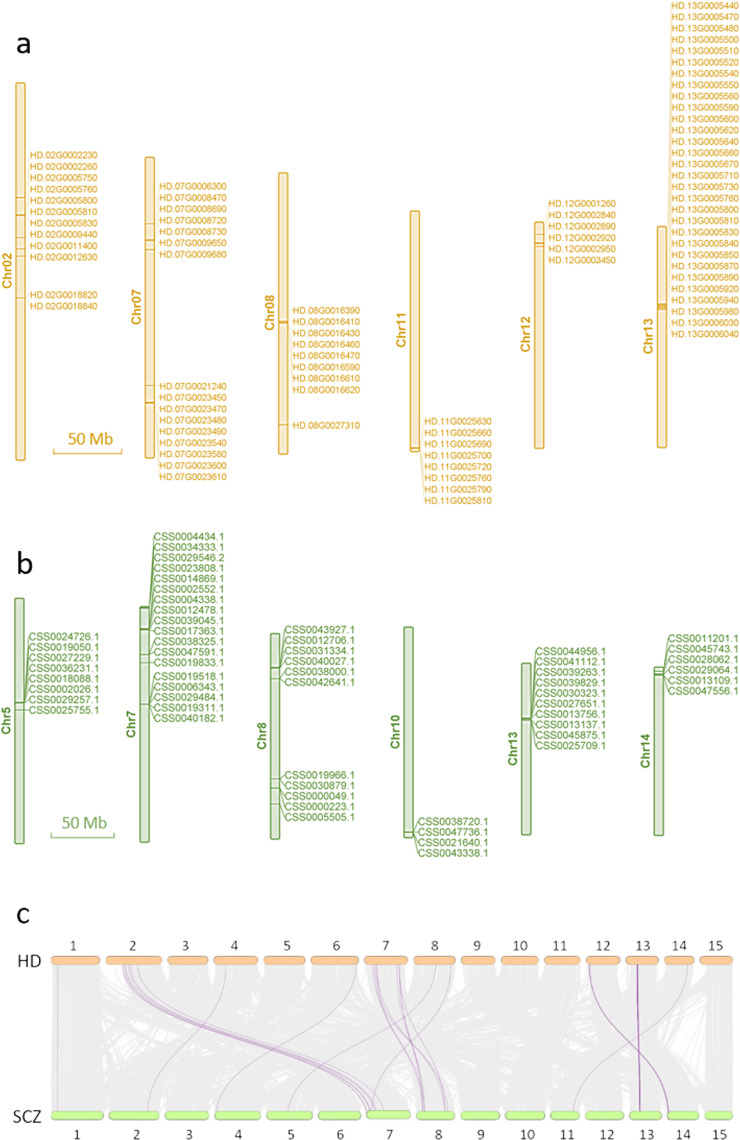
Comparison of TPS genes between HD and SCZ. **a** The TPS genes of HD were mainly distributed on six chromosomes. **b** The TPS genes of SCZ were mainly distributed on six chromosomes. **c** Collinearity analysis of HD and SCZ, in which the purple line indicates the collinearity of TPS genes

As a famous high-aroma backbone parent, HD has been used to breed a series of high-quality offspring. Compared with ordinary tea cultivars, the molecular mechanism of high-aroma formation is worthy of in-depth exploration. Our previous research^24^ showed that the most important aroma components of HD leaves are monoterpenoids and sesquiterpenoids, such as linalool and its oxides, geraniol, nerolidol, and α-farnesene, which are the quality basis for HD that is suitable for processing oolong tea. TPS is an important enzyme that can catalyze the production of a variety of terpenoids, which are essential for the tea aroma from the substrate. Compared with coffee and cacao, the newly released CSS-Suchazao (SCZ) genome showed that the amplification of *TPS* genes was driven by recent tandem replication events^15^. Here, we identified 93 *HDTPS* genes in the HD genome (Supplementary Table 8). This number is greater than the number identified in the SCZ genome (72 *SCZTPSs*)^15^, suggesting that *TPS* genes have expanded considerably in the HD genome. Obviously, compared to *Arabidopsis*, kiwifruit, and coffee (Supplementary Fig. 4), the *TPSs* expanded with tandem duplication were specific to tea plants. *HDTPSs* were widely distributed on 14 chromosomes (Supplementary Table 8); however, they showed a similar clustered pattern and high collinearity across six chromosomes compared with *SCZTPSs* (Fig. 5). Notably, 30 *HDTPSs* were enriched on chromosome 13, and this number was substantially higher than that in the SCZ genome.

**Fig. 6:**
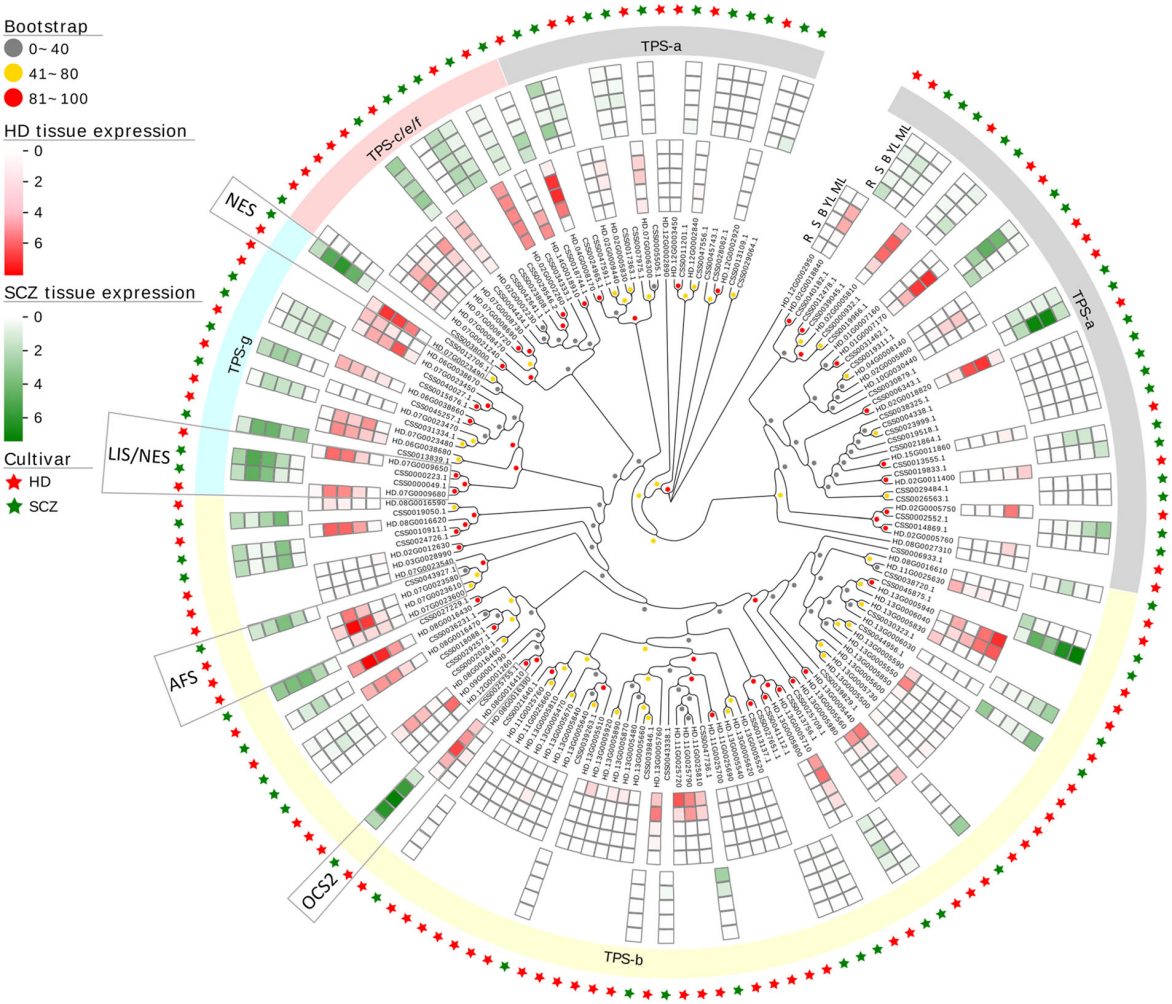
Phylogenetic and tissue expression analysis of TPS genes from HD and SCZ. The legends are shown on the left. The TPM values of five tissues (root, stem, apical bud, young leaf, and mature leaf) from HD and SCZ were normalized. Four homologous TPS genes, including linalool and (*E*)-nerolidol synthase (LIS/NES)^34^, (*E*)-nerolidol synthase (NES)^35^, α-farnesene synthase (AFS)^36^, and ocimene synthase 2 (OCS2)^37^, that have been identified in vitro or in vivo are marked

To compare the transcription abundance of *TPS* genes in HD and SCZ tissues, we mapped the transcriptome data of the five tissues (root, stem, apical bud, young leaf, and mature leaf) to their respective genomes using the same methods aforementioned. Phylogenetic analysis divided *HDTPSs* into four groups (Fig. 6). TPS-b contained the most genes (55, 59.14%), followed by TPS-a (22, 23.66%). Classification of other plants showed that TPS-a is mainly composed of sesquiterpene synthases, TPS-b and TPS-g are mainly monoterpene synthases, and TPS-c/e/f include diterpene synthases^33^. Our results showed that 27 *HDTPSs* were highly expressed in at least one tissue (TPM > 10), whereas 12 were expressed in SCZ (Supplementary Table 8). The apical buds and young leaves of tea plants are the major economically important tissues used for processing tea beverages. We further analyzed the expression of *TPS* genes in buds and young leaves and discovered that 18 *TPSs* were highly expressed in HD (TPM > 10) and 10 in SCZ. We also compared four homologous *TPS* genes, including linalool and (*E*)-nerolidol synthases (LIS/NES, HD.07G0009650, HD.07G0009680, CSS0000049.1, and CSS0000223.1)^34^, (*E*)-NES (HD.07G0023490 and CSS0012706.1)^35^, α-farnesene synthase (AFS, HD.07G0023580, HD.07G0023600, HD.07G0023610, and CSS0043927.1)^36^, and ocimene synthase 2 (OCS2, HD.08G0016410 and CSS0025755.1)^37^, that have been identified in vitro or in vivo (Fig. 6). Among them, the expression levels of the LIS/NES homologous transcripts of HD and SCZ were similar, whereas the expression level of *SCZOCS2* in buds and leaves was higher than that of *HDOCS2*. However, the TPM values of the apical buds and young leaves of one *HDAFS* were 6-fold and 80-fold greater than in SCZ, respectively. The expression of NES of HD in young leaves was nearly 3-fold that of SCZ. In addition, we tested the aroma components of the leaves of HD and SCZ in October. The changes in the marked terpenoids were consistent at the genetic level, except for (*E*)-nerolidol, which may be due to the season and the unprocessed state of the tissues (Supplementary Fig. 5). These results indicate that the widespread and specifically high expression of *TPS* genes in HD tissues may be an important factor affecting their high-aroma characteristics.

### Identification and comparison of C-repeat binding factors (CBFs)

**Fig. 7:**
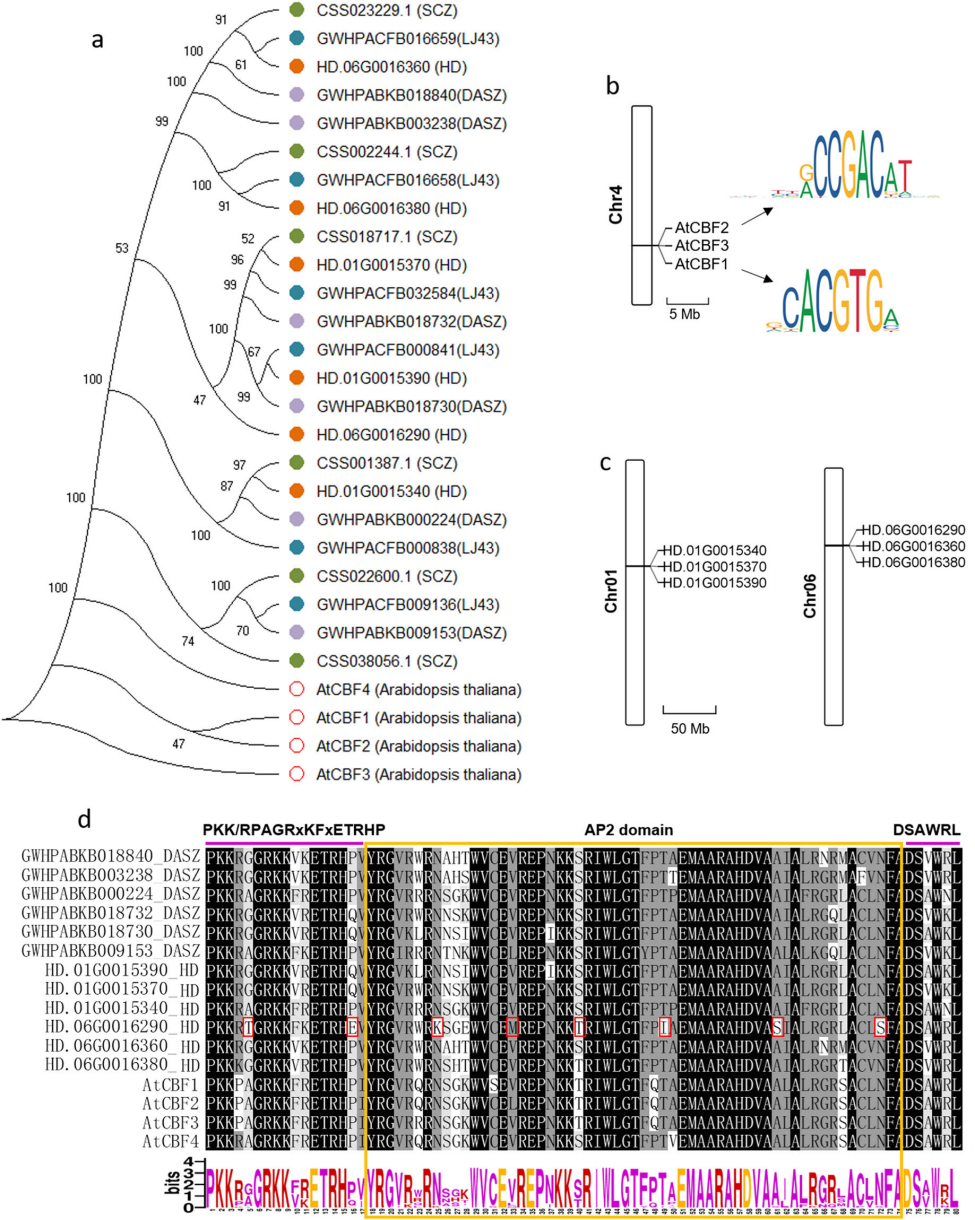
Comparison of C-repeat binding factors (CBFs). **a** Phylogenetic tree analysis of CBFs from four tea resources and *Arabidopsis thaliana*. HD: Huangdan, SCZ: Suchazao^15^, LJ43: Longjing 43^17^, DASZ: An ancient tea tree^19^. **b** Three *AtCBFs* arranged in tandem on chromosome 4 in *Arabidopsis*. The binding sites of *AtCBF1* and *AtCBF2* verified by DNA affinity purification sequencing (DAP-seq) are presented in the form of sequence logos^43^. **c** Six CBFs arranged in tandem on chromosomes 1 and 6 in the HD genome. **d** Multisequence alignment of the conserved binding domains of CBFs from HD, DASZ, and *Arabidopsis*. The sequence logo reflects the conservation of the amino acid at each position. The yellow box represents the AP2 DNA-binding domain; the purple overlines indicate two signature sequences; the small red box represents the amino acid mutation of CBFs of HD compared to DASZ

C-repeat binding factors (CBFs) are the core regulators in response to cold stress in plants; they can be rapidly induced by cold and accompany the C-repeat/dehydration response motif (CRT/DRE) in the promoter region of the target cold-response (COR) genes^38–40^. The CBFs of tea plant were initially identified in the previous draft genome and were found to be rapidly induced by cold stress and to regulate target genes in multiple pathways^41,42^. We are interested in the number of CBF regulatory factors in tea plants, and the recently published chromosome-level genomes of tea plants provide convenience for our identification and comparison. We identified six CBFs in the four tea plant genomes, two more than in *Arabidopsis*, which was consistent with previous experimental verification^42^. The CBFs of tea plant and *Arabidopsis* clustered into different clades, indicating species conservation (Fig. 7a). However, the CBF clades from the four tea germplasms were different, which may be related to the different degrees of cold resistance of various tea plant resources. *Arabidopsis AtCBF1-3* is recognized as an important cascade core that regulates cold acclimation. They are arranged in tandem on chromosome 4 to regulate target genes in response to cold stress through binding G/ACCGAC motifs (Fig. 7b)^40,43^. Interestingly, we observed that the six CBFs in the HD genome were arranged tandemly on chromosomes 1 and 6 (Fig. 7c), which may play an important role in the regulation of cold response in tea plants. Tea plants are believed to originate from southwestern China, and the recently sequenced ancient tea plant (DASZ) genome^19^ from Yunnan Province allows us to compare the evolution and variation of the conserved domain of CBFs. By comparing the AP2 DNA-binding domain and two flanking signature sequences of CBFs among HD, DASZ, and *Arabidopsis*, we observed that the overall CBF protein sequences were highly conserved, and the similarity reached 89.77% (Fig. 7d). Among them, CBFs from tea plants and *Arabidopsis* showed some amino acid differences at multiple positions. Compared with the ancient tea tree, one of the CBF genes (HD.06G0016290) from the elite tea cultivar HD showed amino acid mutations at 8 positions, which may affect its cold response and DNA-binding function. In summary, these results provide genomic evidence for CBF-mediated resistance to cold stress in tea plants.

## Discussion

Oolong tea is one of six major types of tea production and is known for its high levels of aroma^23^. Our study decoded the fully phased genome of an elite oolong tea cultivar, HD. Analysis of the genome revealed extensive genetic variations compared with other published tea genomes and frequent allelic imbalances associated with or likely contributing to high-aroma characteristics and stress tolerance. In addition, we observed a tandem evolution pattern of CBF genes, indicating their potential roles in the stress tolerance.

Aroma plays a vital role in the quality of tea, and in addition to the tea manufacturing process, tea cultivars are the basis for the formation of tea aroma. HD is always well known for its high-aroma characteristics. Our team has participated in the breeding project with HD and CSS Tieguanyin as the backbone parents and has cultivated a series of high-aroma and high-quality offspring, including CSS Jinguanyin (JGY, No. GS2002017), CSS Huangguanyin (HGY, No. GS2002015), and CSS Jinmudan (JMD, No. GS2010024), which are certified by the Chinese Crop Variety Approval Committee as one of the most important pedigrees in Chinese tea breeding^19^. Our previous study showed that sesquiterpenes and monoterpenes are the core aroma components of HD and its progeny^24^. Terpenoids are an important component of tea aroma due to their pleasant aroma and low odor threshold^44^. Comparative genomics of HD and two green tea cultivars revealed extensive structural variations in TPS genes, which may affect the high-aroma characteristics of oolong tea cultivars. The number of TPS genes in SCZ (72) is notably higher than that in coffee (53), cacao (36), and kiwifruit (34)^15^. Our results showed that HD has 21 more TPS genes than SCZ, and they are mainly clustered on six chromosomes, especially chromosome 13, which may enhance the synthesis of terpenoids in HD. Remarkably, compared to SCZ, the TPS genes in HD were more widely and highly expressed in buds and leaves, which are economically important tissues. As a recent study has revealed, the increase in TPS members, the formation of clusters, the differentiation of catalytic functions, and differences in in gene expression regulation may all affect the production and concentration of terpenes in plants^45^. Although the number of TPS genes in tea plants has expanded to varying degrees, the function of most TPS genes is still unknown^46^, except for several TPSs that have been shown to be involved in the formation of linalool^34^, (*E*)-nerolidol^34,35^, (*E*)-β-ocimene^37,47^, and α-farnesene^36,47^. Oolong tea has more pleasant or distinctive aroma qualities than green tea^48^, possibly due to higher levels of (*E*)-nerolidol and α-farnesene^35,49^. This is likely attributable to the significantly upregulated expression of two TPS genes (NES and AFS). Overall, our results suggest that the expansion and specifically high expression of TPS family genes are the molecular basis of HD’s high-aroma characteristics.

## Materials and methods

### Tea plant materials and sequencing

Fresh leaves of an individual CSS cultivar HD planted in the tea plantation of Fujian Agriculture and Forestry University (Fuzhou, China; 26°08′19″ N, 119°24′06″ E) were collected for genome sequencing. The high-quality genomic DNA of HD samples was extracted using a DNeasy Plant Mini Kit (Qiagen, USA) and sent to Annoroad (Ningbo, China) to construct 10–25 kb SMRTbellTM libraries and sequenced using the PacBio Sequel II platform. The tender leaves of HD were also plucked and sent to Annoroad for Hi-C library construction and sequencing through an Illumina HiSeq Nova-seq system. In addition, five HD tissues, including tender roots, young stems, apical buds, tender leaves, and mature leaves, were collected, and the total RNA was extracted by an RNAprep Pure Plant kit (TIANGEN, Beijing, China). A total of 3 μg RNA was used for each sample to generate a transcriptome library and sequenced using the Illumina platform.

### Genome assembly

We analyzed the genome size of the sequenced individuals by flow cytometry (BD FACSCalibur, BD Bioscience, USA) using tomato and maize as internal controls^50^. The CSS HD genome was assembled by incorporating sequencing data from PacBio circular consensus sequencing technology^51^ and the Hi-C method. First, HiFi reads were assembled with Hifiasm with the default parameters. We assembled two levels of chromosome-scale genomes, including a monoploid genome and an allele-defined haplotype-resolved assembly. Briefly, for monoploid assembly, although Purge_dups was contained in Hifiasm, we checked the read depth and filtered the primary contigs of the initial Hifiasm assembly by Purge_dups (v1.25) with the default parameters and evaluated the results by the Benchmarking Universal Single-Copy Orthologs (BUSCO) completeness^27^ and duplication score. Hi-C reads were assessed by the HiC-Pro (v2.11.4) program^52^ and were uniquely mapped to contig assemblies. Meanwhile, Juicer tools (v1.11.08)^53^ and 3D-DNA pipelines (v180114)^54^ were used to detect and correct misassembled contigs. To distribute contigs into the appropriate groups, we aligned the set of Hi-C corrected contigs against the “Shuchazao” chromosome-scale assembly using RaGOO (v1.1). Finally, the ALLHiC optimize algorithm (v0.9.13) was used to adjust the order and orientation of contigs in each group. We merged the haplotig sequence and alternative assembly to redo Purge_dups (v1.25) with the cutoff parameter “2 6 10 12 20 70”. The purged primary contigs and alternative contigs were merged and regarded as a draft contig assembly for the monoploid genome. The resulting contigs were subjected to haplotype phasing using the ALLHiC algorithm with default parameters, and the monoploid genome sequences were selected as a reference to identify allelic contigs. Chromosome-level haplotype A and haplotype B of CSS HD were fully resolved and released. In addition, we calculated the heterozygosity through GenomeScope2^55^ with 33 k-mers. Chromosome localization and collinearity analysis of genes were visualized in TBtools^56^.

### Repetitive sequences and gene annotation

We first ran RepeatModeler (v1.08), which can automatically execute RECON^57^ and RepeatScout^58^, to construct a de novo repeat library of the HD genome. RepeatMasker (v4.07) was utilized to identify and cluster repetitive sequences, and Teclass (v2.1.3)^30^ was used to further classify unknown repetitive sequences. The TRF package (v4.09)^59^ with modified parameters (“trf 1 1 2 80 5 200 2000”) from previous research^4^ was used to find tandem repeats.

We ran two rounds of the MAKER pipeline^29^, which can integrate predictions based on orthologous proteins, ab initio calculations and transcriptome data to better annotate protein-coding genes. We first assembled transcriptome data through de novo assembly and genome-guided assembly by Trinity (v2.20)^60^. Then, the assembled transcripts were subjected to calculation and proper filtering of FPKM expression values with the same standards as in previous research^4^. The filtered data were imported into the PASA program (v2.1.0)^61^ and compared with the UniProt plant protein database for evaluation. Finally, the remaining transcripts were further trained^4^. The evidence was integrated by the MAKER pipeline and used to generate a preliminary set of HD-encoding genes. The predicted gene models with low AED scores were selected for retraining using GENEMARK^62^, SNAP (https://github.com/KorfLab/SNAP)^63^, and AUGUSTUS (v3.3.1)^64^ in the second round of MAKER running. Additionally, the transcriptome data were mapped to the genome via HISAT2 (v2.10) and reassembled via StringTie (v1.3.4)^65^. The assembled data were imported into the MAKER pipeline together with the homologous proteins of *Arabidopsis thaliana*, rice, grape, tomato, *Morus notablis*, and papaya. After filtering putative transposon-derived gene models, a set of HD protein-coding genes was generated. Then, the selected protein-coding gene set was functionally annotated with high-confidence orthologs in the eggNOG database^66^.

### Allele gene and genetic variation identification

Initially, we annotated the HD A/B subgenome with the MAKER pipeline described above. The identification of alleles was based on the methods of sugarcane genome research. We first produced a table of alleles with two columns (including HA and HB). Two main approaches, including the synteny-based approach and the GMAP-based approach^67^, were used to generate the backbone of the allelic table. Initially, we used JCVI (https://github.com/tanghaibao/jcvi)^68^, the Python version of MCSCAN, to scan synteny blocks between two sets of genes. The cscore = 0.99 parameter enabled us to obtain 1 vs. 1 comparison genes between two sets of haplotypes, while genes from the same synteny block were considered alleles to be classified. Subsequently, the gene models excluded from the synteny block were aligned to the monoploid assembly with GMAP (version 2013-10-28). If gene pairs share more than half of the reference genome coordinates, they can exist as alleles. The unfiltered alleles were compared in pairs using the mafft (v7.471) L-Ins-I model to enable accurate alignment between gene pairs. By comparing the alignment results, we were able to calculate the similarity between alleles. Only gene pairs with high similarity (>0.7) were considered to form a pair of alleles, while a pair of identical genes was considered to be the same allele. In the other case, there were multiple genes (>2) aligned to the same location using GMAP. We sorted these genes according to their similarity, removed the gene pairs with low similarity, and added the filtered genes as paralogs of alleles to the allele table.

To explore the genetic difference and evolutionary relationship between two sets of haplotypes, we identified SNPs and InDels between them. SNP identification was performed by the Nucmer utility^69^ with parameter settings consistent with the generation of pseudohaplotypes. The InDels between two subgenomes were found by Assemblytics (http://assemblytics.com/)^70^ with the default parameters. The analysis of structural variants (SVs) among tea plant genomes was also carried out by Nucmer^69^ and Assemblytics^70^.

### Analysis of allele expression

A pipeline built into Trinity software^60^, including bowtie, RNA-Seq by Expectation Maximization (RSEM) and edgeR, was used to analyze the difference in the expression of each pair of alleles in the allele table. In detail, for RNA-seq sequencing reads in five tissues, bud, root, stem, young leaf and mature leaf, we used trim-galore to remove their adaptations before mapping. Subsequently, reads were mapped to allelic transcripts, and the parameters “--all --best --strata -- M 300 --k 1” were used to obtain the optimal results. Then, RSEM was used to calculate the allele transcripts per kilobase of exon model per million mapped reads (TPM) values, and edegR was used to quantitatively evaluate the allelic differences of expression level.

### Analysis of volatile compounds (VOCs)

We collected the leaves of HD and SCZ in October, and the samples were ground to a powder in liquid nitrogen. One gram of the powder was transferred to a 20 ml headspace vial. The vials were sealed using crimp-top caps with TFE-silicone headspace septa. At the time of SPME analysis, each vial was placed at 60 °C for 10 min, and then a 65 µm divinylbenzene/carboxene/polydimethylsilioxane fiber (Supelco, Bellefonte, PA, USA) was exposed to the headspace of the sample for 20 min at 60 °C. VOCs were detected by MetWare (http://www.metware.cn/) based on the Agilent 7890B-7000D platform. Desorption of the VOCs from the fiber coating was carried out in the injection port of the GC apparatus at 250 °C for 5 min in splitless mode. The identification and quantification of VOCs was carried out with a 30 m x 0.25 mm × 1.0 µm DB-5MS (5% phenyl-polymethylsiloxane) capillary column. Helium was used as the carrier gas at a linear velocity of 1.0 ml/min. The oven temperature was programmed from 40 °C (5 min), increased at 6 °C/min to 280 °C, and held for 5 min. Mass spectra were recorded in electron impact (EI) ionization mode at 70 eV. The quadrupole mass detector, ion source and transfer line temperatures were set at 150, 230, and 280 °C, respectively. Mass spectra were scanned in the range *m*/*z* 30–350 amu at 1 s intervals. Volatile compounds were identified by comparing the mass spectra with the data system library (MWGC) and linear retention index.

## Supplementary information

Figure S1-S5

Table S1-S8

## Data Availability

All data are publicly available in the BIG Data Center (https://bigd.big.ac.cn/) under project number PRJCA003382. The raw genome and transcriptome sequencing data are publicly available under sub-number CRA003208. The assembly and annotation of the HD diploid genome are available under sub-number GWHAZTZ00000000. The assembly and annotation of the HD haplotype genome are available under sub-number GWHBAUV00000000.
