# Supplementary material for: Genetic basis of high aroma and stress tolerance in the oolong tea cultivar genome
Source: Hortic Res. 2021 May 1;8:107. doi: 10.1038/s41438-021-00542-x (PMC8087695; doi:10.1038/s41438-021-00542-x)
Supplement: Supplementary file 1 — Figure S1-S5 [file 41438_2021_542_MOESM1_ESM.docx]

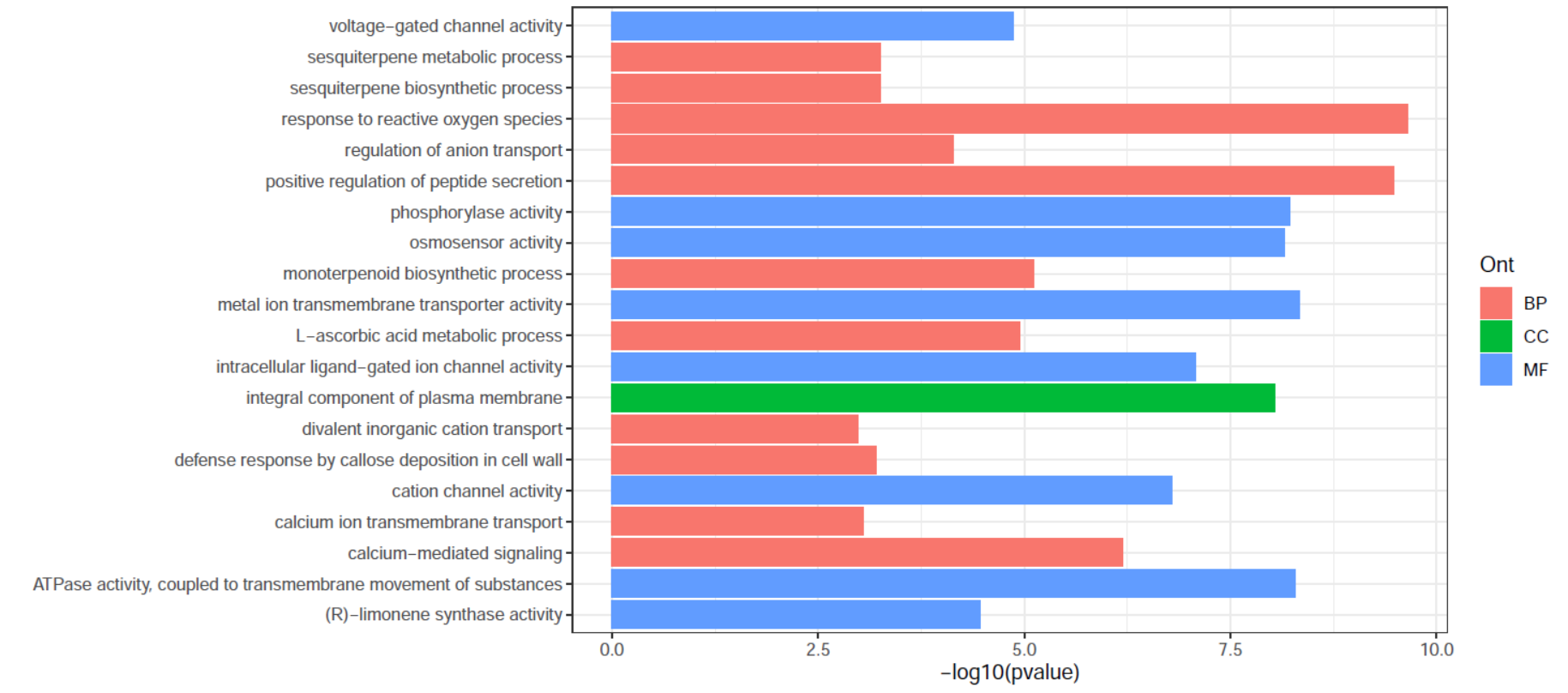


**Supplementary Figure 1. GO enrichment analysis of potential genes under positive selection.**


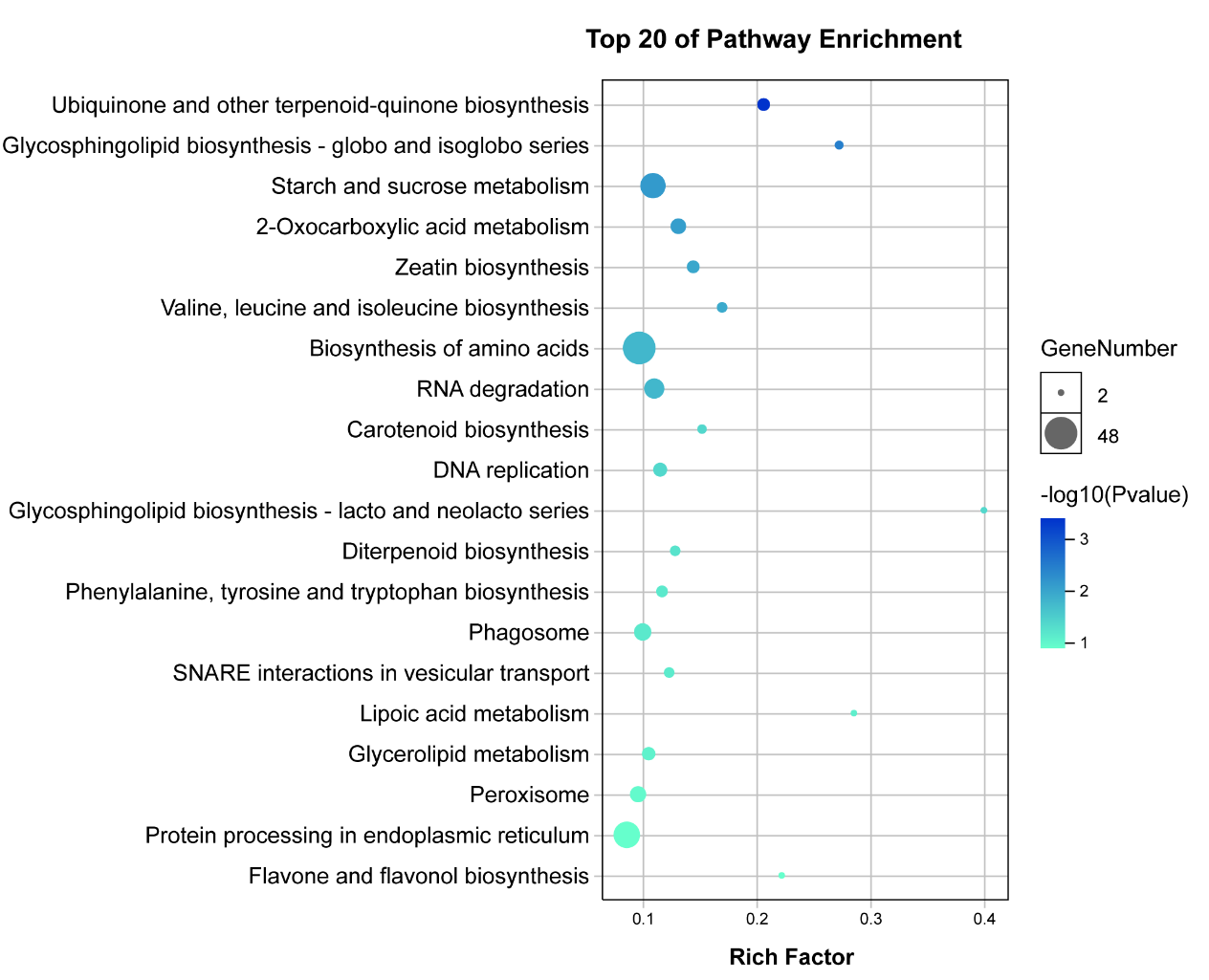


**Supplementary Figure 2. Top 20 KEGG pathways of SV annotated genes shared among three tea plant genomes**

**
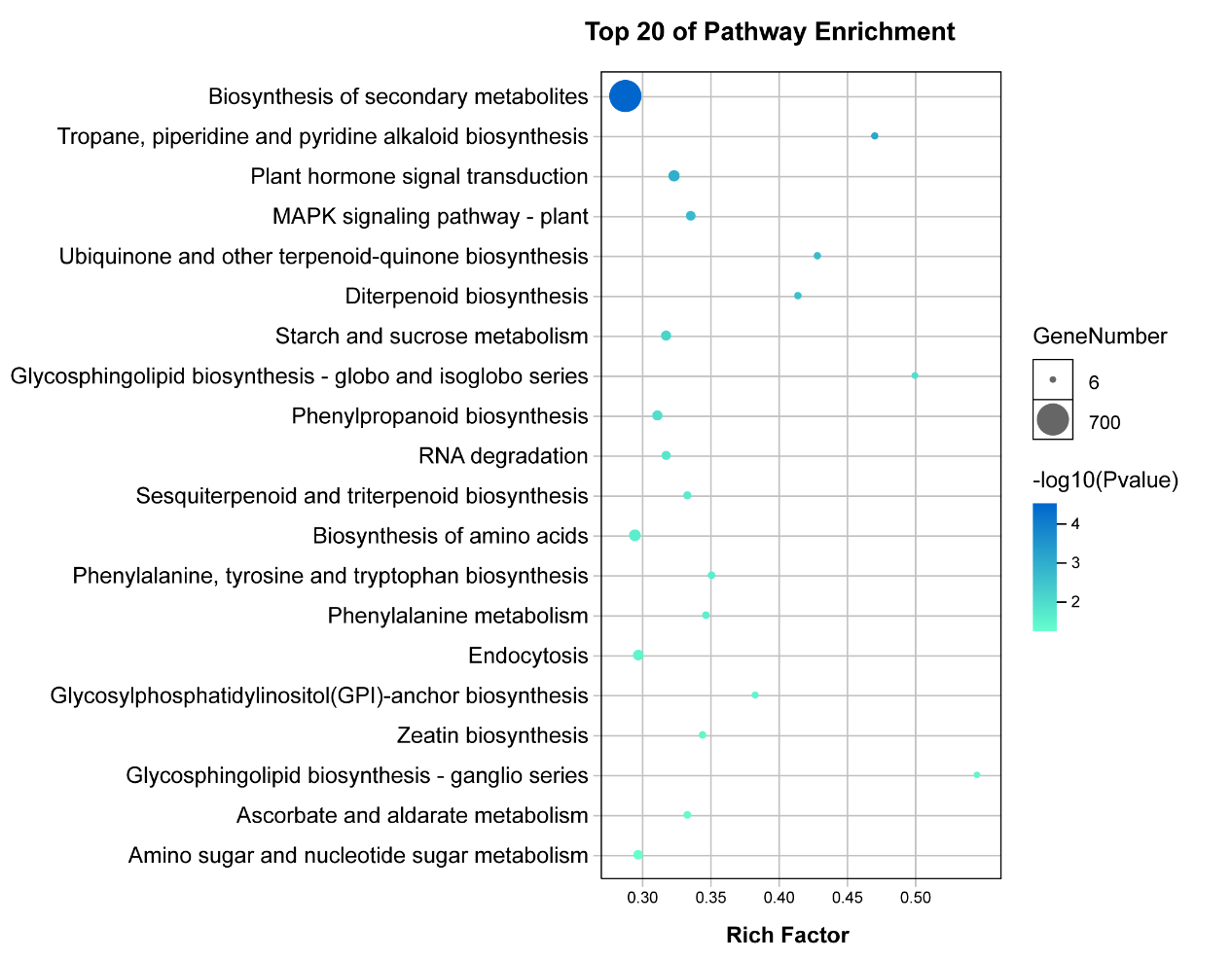
**

**Supplementary Figure 3. Top 20 KEGG pathways of SV annotated genes between HD and LJ43 genome**


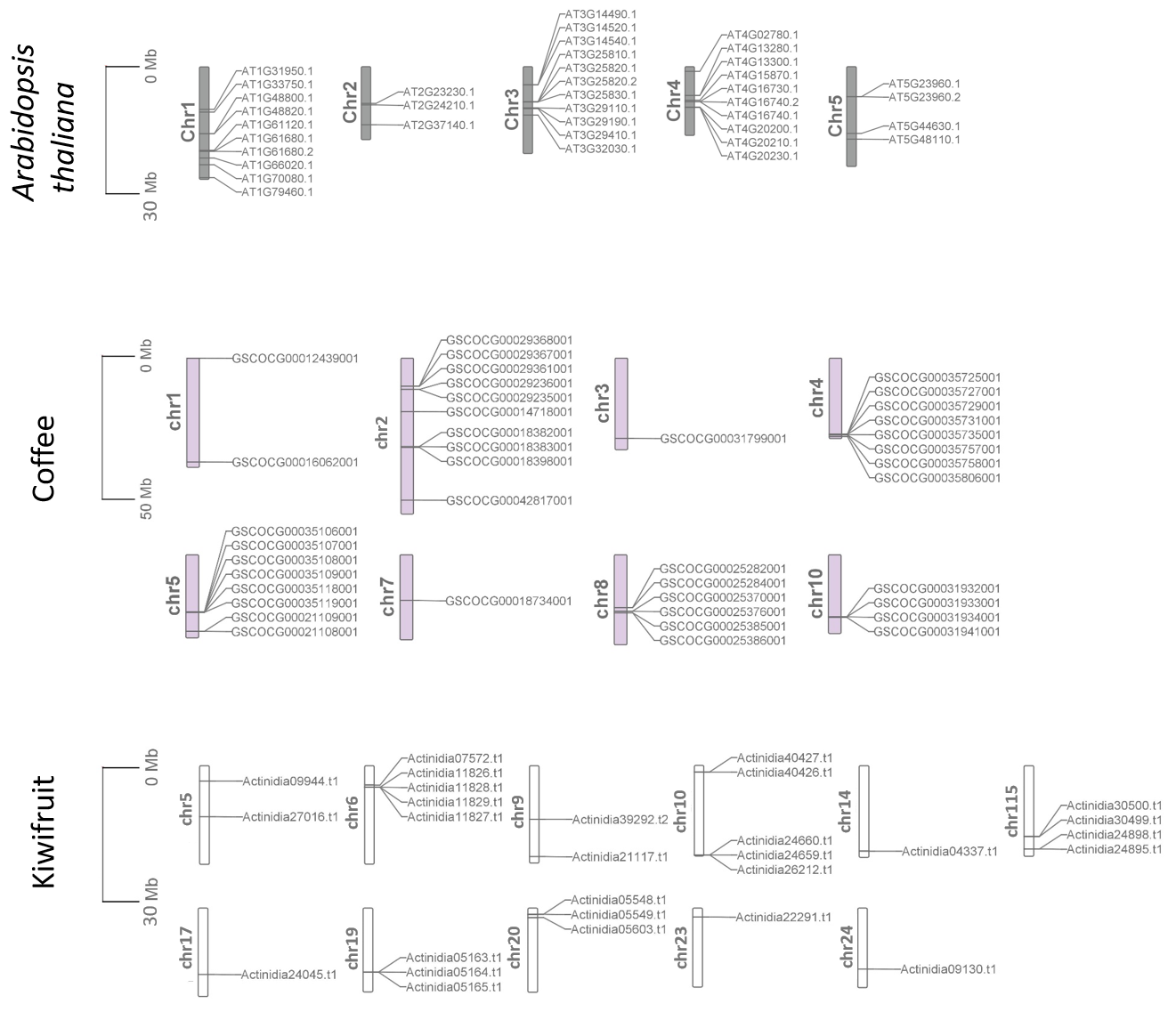


**Supplementary Figure 4. Chromosomal distribution of *TPSs* in *Arabidopsis*, kiwifruit and coffee.**


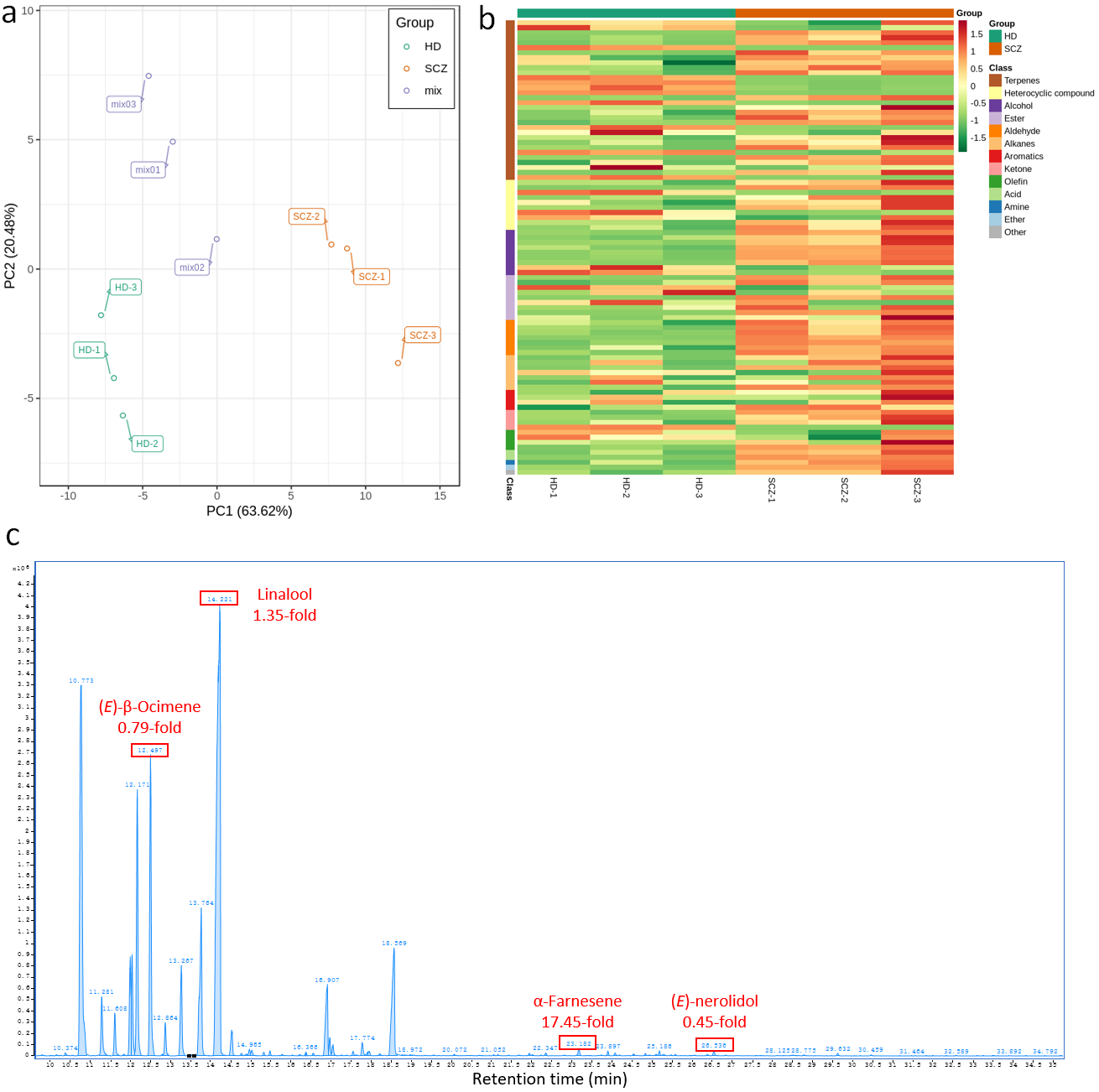


**Supplementary Figure 5. Analysis of volatile compounds (VOCs) between the leaves of HD and SCZ. a** PCA score plot of VOCs between the leaves of HD and SCZ. **b** Heatmap of VOCs. c Total ion chromatogram diagram of VOCs in mix group, where the fold changes (HD/SCZ) of 4 key VOCs are marked.
