# Supplementary material for: Genetic basis of high aroma and stress tolerance in the oolong tea cultivar genome
Source: Hortic Res. 2021 May 1;8:107. doi: 10.1038/s41438-021-00542-x (PMC8087695; doi:10.1038/s41438-021-00542-x)
Supplement: Supplementary file 2 — Table S1-S8 [file 41438_2021_542_MOESM2_ESM.docx]

**Supplementary Table 1. Statistics of Hi-C mapping of the HD genome**

| **Statistics of mapping** | |
| --- | --- |
| Clean Paired-end Reads | 1092569398 |
| Unmapped Paired-end Reads | 53298849 |
| Unmapped Paired-end Reads Rate (%) | 4.878 |
| Paired-end Reads with Singleton | 194205807 |
| Paired-end Reads with Singleton Rate (%) | 17.775 |
| Multi Mapped Paired-end Reads | 0 |
| Multi Mapped Ratio (%) | 0.0 |
| Unique Mapped Paired-end Reads | 336383580 |
| Unique Mapped Ratio (%) | 30.788 |
| **Statistics of valid reads** | |
| Unique Mapped Paired-end Reads | 336383580 |
| Dangling End Paired-end Reads | 16408532 |
| Dangling End Rate (%) | 4.878 |
| Self Circle Paired-end Reads | 2804687 |
| Self Circle Rate (%) | 0.834 |
| Dumped Paired-end Reads | 23787 |
| Dumped Rate (%) | 0.007 |
| Interaction Paired-end Reads | 277379863 |
| Interaction Rate (%) | 82.459 |
| Lib Valid Paired-end Reads | 219862259 |
| Lib Valid Rate (%) | 79.3 |
| Lib Dup (%) | 20.7 |

| Reference | Fluorescence intensity  of reference | Fluorescence intensity  of the sample | Ratio | size (Gb) | average (Gb) | SD |
| --- | --- | --- | --- | --- | --- | --- |
| tomato | 44.78 | 144.05 | 3.22 | 2.83 | 2.94 | 0.10 |
| tomato | 44.60 | 147.74 | 3.31 | 2.92 |  |  |
| tomato | 44.92 | 148.83 | 3.31 | 2.92 |  |  |
| maize | 105.12 | 140.77 | 1.34 | 3.08 |  |  |

**Supplementary Table 2. Flow cytometry to determine the genome size of HD.**

**Supplementary Table 3. BUSCO analysis of annotation completeness of HD genome**

| **Description** | ***HD*** | |
| --- | --- | --- |
|  | **Number** | **Percentage (%)** |
| Complete BUSCOs(C) | 1307 | 95.0 |
| Complete and single-copy BUSCOs(S) | 1191 | 86.6 |
| Complete and duplicated BUSCOs(D) | 116 | 8.4 |
| Fragmented BUSCOs(F) | 19 | 1.4 |
| Missing BUSCOs(M) | 49 | 3.6 |
| Total BUSCO groups searched | 1375 | 100.0 |

**Supplementary Table 4. Statistics of chromosomal-level monoploid assembly of HD**

| **ChrID** | **No. of contigs** | **Length (bp)** |
| --- | --- | --- |
| Chr1 | 1180 | 247215687 |
| Chr2 | 1538 | 219528706 |
| Chr3 | 1344 | 213274022 |
| Chr4 | 1051 | 224367352 |
| Chr5 | 1338 | 227183416 |
| Chr6 | 1456 | 217206614 |
| Chr7 | 1347 | 217509173 |
| Chr8 | 1401 | 211526074 |
| Chr9 | 1394 | 191746340 |
| Chr10 | 1442 | 183612571 |
| Chr11 | 860 | 140763919 |
| Chr12 | 805 | 165738185 |
| Chr13 | 1187 | 159535468 |
| Chr14 | 1054 | 168223539 |
| Chr15 | 1203 | 156641268 |
| Total No. of contigs | 19498 | |
| Total length of contigs (Gb) | 2.94 | |
| Total No. of anchored contigs | 18600 | |
| Total length of chromosome level assembly (Mb) | 2.94 | |
| Anchor rate (%) | 99.83 | |

**Supplementary Table 5. Statistics of intact LTRs identified by LTR_retriever**

| **Genome** | **Superfamily** | **TE type** | **Number of intact LTR** | **Total** |
| --- | --- | --- | --- | --- |
| YK 10 | *Gypsy* | LTR | 2,094 | 3,881 |
|  | *Copia* | LTR | 847 |  |
|  | *unknown* | LTR | 940 |  |
| SCZ-Sca | *Gypsy* | LTR | 2,109 | 4,955 |
|  | *Copia* | LTR | 1,422 |  |
|  | *unknown* | LTR | 1,424 |  |
| SCZ-Chr | *Copia* | LTR | 5,191 | 22,917 |
|  | *Gypsy* | LTR | 10,221 |  |
|  | *unknown* | LTR | 7,505 |  |
| HD | *Gypsy* | LTR | 12,256 | 24,741 |
|  | *Copia* | LTR | 5,145 |  |
|  | *unknown* | LTR | 7,340 |  |

**Supplementary Table 6. TE annotation of three tea plant genomes**

|  | **YK 10** | | **SCZ-Sca** | | | **HD** | |
| --- | --- | --- | --- | --- | --- | --- | --- |
|  | **Length(Mb)** | **% of genome** | **Length(Mb)** | | **% of genome** | **Length(Mb)** | **% of genome** |
| **Total repeat fraction** | 1899.12 | 62.86 | 2071.55 | 65.94 | | 2087.98 | 71.02 |
| **Class I: Retroelement** | 1725.63 | 57.12 | 1759.99 | 56.02 | | 1752.85 | 59.56 |
| **LTR Retrotransposon** | 1389.64 | 46.00 | 1346.94 | 42.88 | | 1422.66 | 48.39 |
| Ty1/Copia | 103.80 | 3.44 | 137.67 | 4.38 | | 12.71 | 4.32 |
| Ty3/Gypsy | 750.38 | 24.84 | 677.64 | 21.57 | | 717.06 | 24.39 |
| Other | 535.46 | 17.72 | 531.64 | 16.92 | | 579.18 | 19.68 |
| **Non-LTR retrotransposon** | 219.41 | 7.26 | 275.18 | 8.76 | | 218.07 | 7.41 |
| LINE | 209.09 | 6.92 | 260.97 | 8.31 | | 205.42 | 6.98 |
| SINE | 10.32 | 0.34 | 14.21 | 0.45 | | 12.65 | 0.43 |
| **Unclassified retroelement** | 116.59 | 3.86 | 137.87 | 4.39 | | 110.65 | 3.76 |
| **Class II: DNA transposon** | 317.06 | 10.49 | 424.81 | 13.52 | | 497.66 | 16.91 |
| **TIR** |  |  |  |  | |  |  |
| CMC[DTC] | 10.73 | 0.36 | 19.27 | 0.61 | | 28.54 | 0.97 |
| hAT | 36.29 | 1.20 | 54.32 | 1.73 | | 37.37 | 1.27 |
| Mutator | 27.43 | 0.91 | 28.45 | 0.91 | | 38.55 | 1.31 |
| Tc1/Mariner | 1.32 | 0.04 | 0.00 | 0.00 | | 0.17 | 0.01 |
| PIF/Harbinger | 15.92 | 0.53 | 18.04 | 0.57 | | 45.02 | 1.53 |
| Other | 224.07 | 7.42 | 304.73 | 9.70 | | 347.56 | 11.81 |
| **Helitron** | 7.25 | 0.24 | 26.23 | 0.83 | | 9.71 | 0.33 |
| **Tandem repeats** | 82.35 | 2.73 | 75.39 | 2.40 | | 137.14 | 4.66 |
| **Unknown** | 61.73 | 2.04 | 82.91 | 2.64 | | 64.45 | 2.19 |

**Supplementary Table 7. Statistics of genetic variation between the two haplotypes in the ‘HD’ genome**

| **ChrID** | **No. of SNPs** | **No. of insertions** | **No. of deletions** | **ChrID** | **No. of SNPs** | **No. of insertions** | **No. of deletions** |
| --- | --- | --- | --- | --- | --- | --- | --- |
| Chr01 | 1914691 | 98925 | 99467 | Chr09 | 1747227 | 82284 | 84368 |
| Chr02 | 2110093 | 95609 | 96455 | Chr10 | 1598210 | 75064 | 76075 |
| Chr03 | 1650772 | 75443 | 76695 | Chr11 | 1244052 | 57114 | 57618 |
| Chr04 | 1675435 | 82629 | 83420 | Chr12 | 1323330 | 64483 | 64708 |
| Chr05 | 1701313 | 75380 | 76665 | Chr13 | 1271166 | 62182 | 63079 |
| Chr06 | 1681069 | 86913 | 88754 | Chr14 | 1200791 | 67298 | 67070 |
| Chr07 | 1785067 | 88426 | 90150 | Chr15 | 1147239 | 51796 | 52919 |
| Chr08 | 1526602 | 71418 | 71971 | Total | 23577042 | 1134967 | 1149416 |

| **Supplementary Table 8. The terpene synthase (TPS) genes in HD and SCZ genomes** | | | | | | | |
| --- | --- | --- | --- | --- | --- | --- | --- |
| **Gene ID** | **Chromosome** | **Subfamily** | **Root** | **Stem** | **Aptical bud** | **Young leaf** | **Mature leaf** |
| *HD.01G0007160* | Chr1 | TPS-a | 0.00 | 0.00 | 0.00 | 0.00 | 0.00 |
| *HD.01G0007170* | Chr1 | TPS-a | 0.26 | 3.82 | 73.69 | 94.89 | 0.00 |
| *HD.02G0005750* | Chr2 | TPS-a | 0.00 | 0.10 | 0.00 | 17.52 | 0.00 |
| *HD.02G0005760* | Chr2 | TPS-a | 0.00 | 0.00 | 0.00 | 1.40 | 0.00 |
| *HD.02G0005800* | Chr2 | TPS-a | 0.00 | 0.16 | 1.53 | 2.21 | 0.00 |
| *HD.02G0005810* | Chr2 | TPS-a | 0.00 | 1.47 | 24.85 | 32.97 | 3.42 |
| *HD.02G0005830* | Chr2 | TPS-a | 0.00 | 0.10 | 0.56 | 0.61 | 0.00 |
| *HD.02G0009440* | Chr2 | TPS-a | 0.00 | 0.00 | 0.05 | 0.00 | 0.00 |
| *HD.02G0011400* | Chr2 | TPS-a | 0.00 | 0.00 | 0.07 | 0.31 | 1.90 |
| *HD.02G0018820* | Chr2 | TPS-a | 0.14 | 0.55 | 44.82 | 79.02 | 0.56 |
| *HD.02G0018840* | Chr2 | TPS-a | 0.00 | 0.10 | 4.98 | 4.76 | 0.05 |
| *HD.04G0008140* | Chr4 | TPS-a | 0.00 | 0.00 | 0.00 | 0.00 | 0.00 |
| *HD.04G0008170* | Chr4 | TPS-a | 0.00 | 18.03 | 96.65 | 84.22 | 0.41 |
| *HD.07G0006300* | Chr7 | TPS-a | 0.63 | 0.04 | 2.10 | 1.58 | 0.12 |
| *HD.08G0027310* | Chr8 | TPS-a | 0.00 | 0.00 | 0.00 | 0.00 | 0.00 |
| *HD.10G0030440* | Chr10 | TPS-a | 0.00 | 0.00 | 0.00 | 0.00 | 0.00 |
| *HD.12G0002840* | Chr12 | TPS-a | 0.07 | 0.21 | 0.00 | 0.00 | 0.00 |
| *HD.12G0002890* | Chr12 | TPS-a | 0.00 | 0.00 | 0.00 | 0.00 | 0.00 |
| *HD.12G0002920* | Chr12 | TPS-a | 0.00 | 0.00 | 0.00 | 0.00 | 0.00 |
| *HD.12G0002950* | Chr12 | TPS-a | 0.06 | 0.00 | 0.00 | 0.00 | 0.00 |
| *HD.12G0003450* | Chr12 | TPS-a | 0.00 | 0.00 | 0.00 | 0.00 | 0.00 |
| *HD.15G0011860* | Chr15 | TPS-a | 0.00 | 0.00 | 0.06 | 0.38 | 0.00 |
| *HD.02G0012630* | Chr2 | TPS-b | 0.21 | 0.00 | 0.00 | 0.00 | 0.00 |
| *HD.03G0028990* | Chr3 | TPS-b | 0.00 | 0.00 | 0.00 | 0.00 | 0.00 |
| *HD.07G0023540* | Chr7 | TPS-b | 0.00 | 0.00 | 0.06 | 0.12 | 0.00 |
| *HD.07G0023580* | Chr7 | TPS-b | 0.00 | 0.00 | 2.66 | 16.80 | 0.00 |
| *HD.07G0023600* | Chr7 | TPS-b | 0.00 | 1.09 | 1.16 | 0.84 | 0.00 |
| *HD.07G0023610* | Chr7 | TPS-b | 0.00 | 0.80 | 70.40 | 218.11 | 1.50 |
| *HD.08G0016390* | Chr8 | TPS-b | 0.00 | 0.05 | 0.47 | 0.05 | 0.00 |
| *HD.08G0016410* | Chr8 | TPS-b | 2.18 | 9.22 | 54.70 | 3.96 | 0.19 |
| *HD.08G0016430* | Chr8 | TPS-b | 0.10 | 7.61 | 108.04 | 256.61 | 8.29 |
| *HD.08G0016460* | Chr8 | TPS-b | 0.00 | 0.18 | 3.00 | 6.07 | 0.59 |
| *HD.08G0016470* | Chr8 | TPS-b | 0.00 | 1.18 | 8.84 | 8.71 | 4.15 |
| *HD.08G0016590* | Chr8 | TPS-b | 0.00 | 0.12 | 0.36 | 0.79 | 0.00 |
| *HD.08G0016610* | Chr8 | TPS-b | 0.00 | 0.00 | 0.00 | 0.00 | 0.00 |
| *HD.08G0016620* | Chr8 | TPS-b | 0.16 | 6.50 | 27.70 | 30.43 | 0.00 |
| *HD.09G0001790* | Chr9 | TPS-b | 23.01 | 0.00 | 0.00 | 0.00 | 0.00 |
| *HD.11G0025630* | Chr11 | TPS-b | 4.20 | 0.34 | 0.39 | 0.00 | 0.00 |
| *HD.11G0025660* | Chr11 | TPS-b | 0.00 | 0.00 | 0.00 | 0.00 | 0.00 |
| *HD.11G0025690* | Chr11 | TPS-b | 0.00 | 0.00 | 0.00 | 0.00 | 0.00 |
| *HD.11G0025700* | Chr11 | TPS-b | 0.00 | 0.12 | 0.00 | 0.00 | 0.00 |
| *HD.11G0025720* | Chr11 | TPS-b | 38.68 | 1.05 | 0.00 | 0.00 | 0.00 |
| *HD.11G0025760* | Chr11 | TPS-b | 0.00 | 0.00 | 0.00 | 0.07 | 0.00 |
| *HD.11G0025790* | Chr11 | TPS-b | 13.70 | 8.25 | 0.00 | 0.00 | 0.00 |
| *HD.11G0025810* | Chr11 | TPS-b | 2.52 | 1.57 | 0.00 | 0.00 | 0.00 |
| *HD.12G0001260* | Chr12 | TPS-b | 0.00 | 0.00 | 0.00 | 0.00 | 0.00 |
| *HD.13G0005440* | Chr13 | TPS-b | 16.44 | 0.84 | 0.00 | 0.00 | 2.35 |
| *HD.13G0005470* | Chr13 | TPS-b | 0.00 | 0.00 | 0.00 | 0.00 | 0.00 |
| *HD.13G0005480* | Chr13 | TPS-b | 0.56 | 0.00 | 0.00 | 0.00 | 0.00 |
| *HD.13G0005500* | Chr13 | TPS-b | 0.21 | 0.00 | 0.00 | 0.07 | 0.00 |
| *HD.13G0005510* | Chr13 | TPS-b | 0.00 | 0.00 | 0.00 | 0.00 | 0.00 |
| *HD.13G0005520* | Chr13 | TPS-b | 0.00 | 0.00 | 0.00 | 0.00 | 0.00 |
| *HD.13G0005540* | Chr13 | TPS-b | 0.00 | 0.00 | 0.00 | 0.05 | 0.00 |
| *HD.13G0005550* | Chr13 | TPS-b | 13.61 | 0.09 | 0.00 | 0.00 | 0.00 |
| *HD.13G0005560* | Chr13 | TPS-b | 8.54 | 0.63 | 0.00 | 0.00 | 0.00 |
| *HD.13G0005590* | Chr13 | TPS-b | 1.31 | 0.00 | 0.00 | 0.00 | 0.15 |
| *HD.13G0005600* | Chr13 | TPS-b | 0.00 | 0.00 | 0.00 | 0.00 | 0.00 |
| *HD.13G0005620* | Chr13 | TPS-b | 0.00 | 0.00 | 0.00 | 0.00 | 0.00 |
| *HD.13G0005640* | Chr13 | TPS-b | 0.00 | 0.00 | 0.00 | 0.00 | 0.00 |
| *HD.13G0005660* | Chr13 | TPS-b | 0.00 | 0.00 | 0.08 | 0.00 | 0.00 |
| *HD.13G0005670* | Chr13 | TPS-b | 0.00 | 0.00 | 0.51 | 0.00 | 0.00 |
| *HD.13G0005710* | Chr13 | TPS-b | 7.22 | 19.23 | 1.29 | 0.98 | 0.00 |
| *HD.13G0005730* | Chr13 | TPS-b | 0.00 | 0.00 | 0.00 | 0.00 | 0.34 |
| *HD.13G0005760* | Chr13 | TPS-b | 2.41 | 16.50 | 0.34 | 0.27 | 0.00 |
| *HD.13G0005800* | Chr13 | TPS-b | 0.00 | 0.00 | 0.00 | 0.00 | 0.00 |
| *HD.13G0005810* | Chr13 | TPS-b | 0.00 | 0.00 | 0.00 | 0.00 | 0.00 |
| *HD.13G0005830* | Chr13 | TPS-b | 2.16 | 0.00 | 0.00 | 0.00 | 0.06 |
| *HD.13G0005840* | Chr13 | TPS-b | 0.00 | 0.00 | 0.00 | 0.00 | 0.00 |
| *HD.13G0005850* | Chr13 | TPS-b | 0.32 | 0.16 | 0.00 | 0.00 | 0.15 |
| *HD.13G0005870* | Chr13 | TPS-b | 0.47 | 0.00 | 0.00 | 0.00 | 0.00 |
| *HD.13G0005890* | Chr13 | TPS-b | 0.00 | 0.00 | 0.00 | 0.00 | 0.00 |
| *HD.13G0005920* | Chr13 | TPS-b | 1.48 | 0.00 | 0.00 | 0.00 | 0.00 |
| *HD.13G0005940* | Chr13 | TPS-b | 0.05 | 4.93 | 0.00 | 19.99 | 93.57 |
| *HD.13G0005980* | Chr13 | TPS-b | 0.00 | 0.00 | 0.00 | 0.00 | 0.00 |
| *HD.13G0006030* | Chr13 | TPS-b | 0.00 | 0.00 | 0.00 | 0.00 | 0.07 |
| *HD.13G0006040* | Chr13 | TPS-b | 0.00 | 1.20 | 3.75 | 20.68 | 35.93 |
| *HD.02G0002230* | Chr2 | TPS-c/e/f | 0.40 | 0.67 | 1.53 | 2.82 | 1.25 |
| *HD.02G0002260* | Chr2 | TPS-c/e/f | 10.94 | 12.93 | 12.10 | 11.48 | 7.28 |
| *HD.07G0008470* | Chr7 | TPS-c/e/f | 0.00 | 1.68 | 10.08 | 4.13 | 0.10 |
| *HD.07G0008690* | Chr7 | TPS-c/e/f | 0.00 | 0.00 | 0.00 | 0.00 | 0.00 |
| *HD.07G0008720* | Chr7 | TPS-c/e/f | 0.06 | 0.00 | 0.00 | 0.65 | 0.00 |
| *HD.07G0008730* | Chr7 | TPS-c/e/f | 0.00 | 0.71 | 1.66 | 7.81 | 0.10 |
| *HD.07G0021240* | Chr7 | TPS-c/e/f | 0.00 | 0.39 | 0.00 | 0.00 | 0.00 |
| *HD.14G0018910* | Chr14 | TPS-c/e/f | 10.83 | 5.41 | 0.10 | 0.00 | 0.00 |
| *HD.06G0038660* | Chr6 | TPS-g | 0.00 | 0.74 | 1.14 | 3.02 | 4.05 |
| *HD.06G0038670* | Chr6 | TPS-g | 0.00 | 0.00 | 0.69 | 2.38 | 2.53 |
| *HD.06G0038680* | Chr6 | TPS-g | 0.70 | 2.10 | 5.59 | 11.75 | 0.09 |
| *HD.07G0009650* | Chr7 | TPS-g | 0.00 | 1.61 | 18.08 | 24.35 | 0.13 |
| *HD.07G0009680* | Chr7 | TPS-g | 0.00 | 1.15 | 11.43 | 14.54 | 0.17 |
| *HD.07G0023450* | Chr7 | TPS-g | 0.59 | 38.02 | 4.72 | 5.23 | 0.41 |
| *HD.07G0023470* | Chr7 | TPS-g | 0.00 | 0.00 | 0.00 | 0.00 | 0.00 |
| *HD.07G0023480* | Chr7 | TPS-g | 0.07 | 9.57 | 1.98 | 4.53 | 0.07 |
| *HD.07G0023490* | Chr7 | TPS-g | 0.00 | 14.42 | 61.41 | 79.51 | 0.93 |
| *CSS0000932.1* | Chr8 | TPS-a | 0.00 | 0.17 | 0.30 | 0.16 | 0.00 |
| *CSS0002552.1* | Chr8 | TPS-a | 0.00 | 0.20 | 0.31 | 2.86 | 6.53 |
| *CSS0004338.1* | Chr2 | TPS-a | 0.00 | 0.00 | 0.00 | 0.00 | 0.00 |
| *CSS0005505.1* | Chr5 | TPS-a | 0.02 | 0.02 | 0.06 | 0.04 | 0.02 |
| *CSS0006343.1* | Chr7 | TPS-a | 0.10 | 2.93 | 124.73 | 117.76 | 2.11 |
| *CSS0007975.1* | Chr7 | TPS-a | 0.00 | 0.00 | 0.56 | 0.24 | 0.02 |
| *CSS0011201.1* | Chr7 | TPS-a | 0.28 | 0.10 | 0.02 | 0.04 | 0.04 |
| *CSS0012478.1* | Chr8 | TPS-a | 0.00 | 0.10 | 0.58 | 1.23 | 0.02 |
| *CSS0013109.1* | Chr7 | TPS-a | 0.05 | 0.00 | 0.00 | 0.00 | 0.00 |
| *CSS0013555.1* | Chr3 | TPS-a | 0.00 | 0.13 | 1.90 | 1.46 | 0.06 |
| *CSS0014869.1* | Contig865 | TPS-a | 0.00 | 0.00 | 0.00 | 0.00 | 0.00 |
| *CSS0017363.1* | Contig434 | TPS-a | 0.18 | 0.07 | 0.46 | 0.21 | 0.15 |
| *CSS0019311.1* | Chr14 | TPS-a | 3.30 | 0.56 | 0.04 | 0.09 | 0.09 |
| *CSS0019518.1* | Chr7 | TPS-a | 0.00 | 0.00 | 0.00 | 0.00 | 0.00 |
| *CSS0019833.1* | Chr8 | TPS-a | 0.00 | 0.02 | 0.06 | 0.16 | 1.72 |
| *CSS0019966.1* | Chr14 | TPS-a | 0.04 | 1.75 | 0.00 | 0.08 | 0.52 |
| *CSS0021864.1* | Chr13 | TPS-a | 0.00 | 0.00 | 0.00 | 0.00 | 0.00 |
| *CSS0023999.1* | Chr15 | TPS-a | 0.07 | 0.16 | 0.00 | 0.07 | 0.08 |
| *CSS0024965.1* | Chr13 | TPS-a | 0.00 | 6.10 | 0.40 | 0.08 | 3.71 |
| *CSS0026563.1* | Chr4 | TPS-a | 0.00 | 0.00 | 0.00 | 0.00 | 0.00 |
| *CSS0028062.1* | Chr7 | TPS-a | 0.00 | 0.00 | 0.00 | 0.00 | 0.00 |
| *CSS0029064.1* | Contig1247 | TPS-a | 0.76 | 0.00 | 0.00 | 0.00 | 0.00 |
| *CSS0029484.1* | Chr7 | TPS-a | 0.00 | 0.00 | 0.00 | 0.00 | 0.00 |
| *CSS0030879.1* | Chr5 | TPS-a | 0.00 | 1.36 | 0.42 | 0.60 | 0.76 |
| *CSS0031462.1* | Chr11 | TPS-a | 0.00 | 5.36 | 22.13 | 16.08 | 0.38 |
| *CSS0038325.1* | Chr5 | TPS-a | 0.00 | 0.00 | 0.00 | 0.00 | 0.00 |
| *CSS0039045.1* | Chr7 | TPS-a | 0.00 | 0.02 | 0.13 | 0.16 | 0.05 |
| *CSS0040182.1* | Chr7 | TPS-a | 2.13 | 0.45 | 0.13 | 0.48 | 0.07 |
| *CSS0045743.1* | Chr7 | TPS-a | 0.05 | 0.00 | 0.14 | 0.00 | 0.00 |
| *CSS0047556.1* | Chr8 | TPS-a | 0.15 | 0.11 | 0.00 | 0.00 | 0.00 |
| *CSS0047591.1* | Chr10 | TPS-a | 0.00 | 0.11 | 0.78 | 0.44 | 0.07 |
| *CSS0002026.1* | Chr15 | TPS-b | 0.00 | 0.10 | 0.00 | 0.00 | 0.00 |
| *CSS0006933.1* | Chr7 | TPS-b | 0.00 | 0.15 | 2.08 | 0.14 | 0.00 |
| *CSS0010911.1* | Chr2 | TPS-b | 0.13 | 9.99 | 0.63 | 0.29 | 3.19 |
| *CSS0013137.1* | Chr5 | TPS-b | 0.00 | 0.04 | 0.00 | 0.00 | 0.00 |
| *CSS0013756.1* | Chr2 | TPS-b | 0.82 | 0.42 | 2.18 | 0.61 | 0.00 |
| *CSS0018088.1* | Chr13 | TPS-b | 0.00 | 0.34 | 0.00 | 0.08 | 0.25 |
| *CSS0019050.1* | Chr5 | TPS-b | 0.10 | 15.64 | 4.37 | 0.77 | 2.97 |
| *CSS0021640.1* | Chr15 | TPS-b | 0.00 | 0.00 | 0.00 | 0.00 | 0.00 |
| *CSS0024726.1* | Chr5 | TPS-b | 0.17 | 9.82 | 1.81 | 0.25 | 2.79 |
| *CSS0025709.1* | Chr13 | TPS-b | 0.10 | 0.00 | 0.00 | 0.00 | 0.32 |
| *CSS0025755.1* | Chr14 | TPS-b | 1.44 | 49.63 | 156.57 | 68.41 | 3.38 |
| *CSS0027229.1* | Chr14 | TPS-b | 0.15 | 3.64 | 15.60 | 12.40 | 7.18 |
| *CSS0027651.1* | Chr5 | TPS-b | 0.22 | 0.02 | 0.08 | 0.02 | 0.00 |
| *CSS0029257.1* | Chr7 | TPS-b | 0.00 | 0.25 | 1.11 | 0.31 | 0.08 |
| *CSS0030323.1* | Chr7 | TPS-b | 0.17 | 0.21 | 0.34 | 1.80 | 5.57 |
| *CSS0036231.1* | Chr13 | TPS-b | 0.00 | 0.00 | 2.81 | 0.94 | 0.13 |
| *CSS0038720.1* | Chr8 | TPS-b | 2.98 | 0.15 | 0.00 | 0.00 | 0.00 |
| *CSS0039263.1* | Chr8 | TPS-b | 0.00 | 0.04 | 0.00 | 0.00 | 0.00 |
| *CSS0039829.1* | Chr1 | TPS-b | 0.00 | 0.00 | 0.00 | 0.00 | 6.04 |
| *CSS0039846.1* | Chr7 | TPS-b | 0.00 | 0.00 | 0.00 | 0.00 | 0.00 |
| *CSS0041112.1* | Chr5 | TPS-b | 0.00 | 0.00 | 0.09 | 0.05 | 0.00 |
| *CSS0043338.1* | Chr8 | TPS-b | 0.00 | 0.26 | 0.00 | 0.00 | 0.00 |
| *CSS0043927.1* | Chr7 | TPS-b | 0.00 | 2.41 | 11.70 | 2.72 | 1.39 |
| *CSS0044956.1* | Chr10 | TPS-b | 7.11 | 0.14 | 0.36 | 0.61 | 10.78 |
| *CSS0045875.1* | Chr7 | TPS-b | 0.63 | 21.63 | 8.51 | 84.65 | 159.83 |
| *CSS0047736.1* | Chr13 | TPS-b | 5.16 | 1.21 | 0.00 | 0.00 | 0.04 |
| *CSS0004434.1* | Chr13 | TPS-c/e/f | 0.75 | 6.80 | 3.48 | 2.69 | 5.83 |
| *CSS0018744.1* | Contig693 | TPS-c/e/f | 1.78 | 4.18 | 0.23 | 0.08 | 0.01 |
| *CSS0023808.1* | Chr8 | TPS-c/e/f | 2.03 | 3.74 | 1.90 | 2.69 | 2.55 |
| *CSS0029546.2* | Chr7 | TPS-c/e/f | 2.90 | 1.32 | 0.93 | 0.48 | 0.75 |
| *CSS0034333.1* | Chr13 | TPS-c/e/f | 0.56 | 1.20 | 0.44 | 0.17 | 0.39 |
| *CSS0038000.1* | Chr8 | TPS-c/e/f | 0.00 | 0.00 | 0.00 | 0.00 | 0.00 |
| *CSS0042641.1* | Chr10 | TPS-c/e/f | 0.00 | 0.00 | 0.00 | 0.00 | 0.00 |
| *CSS0000049.1* | Chr8 | TPS-g | 0.07 | 0.87 | 10.64 | 22.72 | 2.45 |
| *CSS0000223.1* | Chr13 | TPS-g | 0.12 | 3.09 | 17.31 | 30.61 | 4.82 |
| *CSS0012706.1* | Chr4 | TPS-g | 0.83 | 19.70 | 62.15 | 27.56 | 2.54 |
| *CSS0013839.1* | Chr14 | TPS-g | 7.51 | 2.67 | 18.90 | 17.33 | 1.14 |
| *CSS0015676.1* | Chr13 | TPS-g | 0.00 | 1.67 | 0.90 | 0.88 | 0.00 |
| *CSS0031334.1* | Chr14 | TPS-g | 0.00 | 1.36 | 1.94 | 2.69 | 0.19 |
| *CSS0040027.1* | Chr7 | TPS-g | 0.10 | 2.26 | 1.18 | 1.02 | 0.10 |
| *CSS0045257.1* | Chr10 | TPS-g | 0.00 | 0.00 | 4.86 | 5.60 | 2.60 |
|  |  |  |  |  |  |  |  |
